# Drug target inference by mining transcriptional data using a novel graph convolutional network framework

**DOI:** 10.1007/s13238-021-00885-0

**Published:** 2021-10-22

**Authors:** Feisheng Zhong, Xiaolong Wu, Ruirui Yang, Xutong Li, Dingyan Wang, Zunyun Fu, Xiaohong Liu, XiaoZhe Wan, Tianbiao Yang, Zisheng Fan, Yinghui Zhang, Xiaomin Luo, Kaixian Chen, Sulin Zhang, Hualiang Jiang, Mingyue Zheng

**Affiliations:** 1 Drug Discovery and Design Center, State Key Laboratory of Drug Research, Shanghai Institute of Materia Medica, Chinese Academy of Sciences, Shanghai, 201203 China; 2 University of Chinese Academy of Sciences, Beijing, 100049 China; 3 School of Pharmacy, East China University of Science and Technology, Shanghai, 200237 China; 4 Nanjing University of Chinese Medicine, Nanjing, 210023 China; 5 Shanghai Institute for Advanced Immunochemical Studies, and School of Life Science and Technology, ShanghaiTech University, Shanghai, 200031 China

**Keywords:** drug target inference, transcriptomics, deep learning, experimental verification

## Abstract

A fundamental challenge that arises in biomedicine is the need to characterize compounds in a relevant cellular context in order to reveal potential on-target or off-target effects. Recently, the fast accumulation of gene transcriptional profiling data provides us an unprecedented opportunity to explore the protein targets of chemical compounds from the perspective of cell transcriptomics and RNA biology. Here, we propose a novel Siamese spectral-based graph convolutional network (SSGCN) model for inferring the protein targets of chemical compounds from gene transcriptional profiles. Although the gene signature of a compound perturbation only provides indirect clues of the interacting targets, and the biological networks under different experiment conditions further complicate the situation, the SSGCN model was successfully trained to learn from known compound-target pairs by uncovering the hidden correlations between compound perturbation profiles and gene knockdown profiles. On a benchmark set and a large time-split validation dataset, the model achieved higher target inference accuracy as compared to previous methods such as Connectivity Map. Further experimental validations of prediction results highlight the practical usefulness of SSGCN in either inferring the interacting targets of compound, or reversely, in finding novel inhibitors of a given target of interest.

**Supplementary Information:**

The online version contains supplementary material available at 10.1007/s13238-021-00885-0.

## Introduction

Because most drugs exert their therapeutic effects by interacting with their *in vivo* targets, target prediction plays a pivotal role in early drug discovery and development, particularly during the era of polypharmacology (Anighoro et al., 2014). In the context of polypharmacology, the “magic bullet” is likely an exceptional case, and *in silico* target prediction can be used to explore the whole therapeutic target space for a given molecule. This procedure might help deepen our understanding of the mechanisms of action, metabolism, adverse effects, and drug resistance of a molecule. By predicting targets of approved drugs, these clinically used chemicals can be repurposed for other diseases (Ashburn and Thor, 2004); for example, sildenafil (Terrett et al., 1996) is used to treat erectile dysfunction but was first developed for the treatment of angina.

Targets of candidate molecules can either be identified via biochemical experiments, such as protein proteomic mass spectrometry, or predicted using computational approaches. Computational target prediction has gained momentum due to its low cost and high-throughput nature. The classical methods generally include ligand-based (Geppert et al., 2010) and structure-based methods (Schomburg et al., 2014) : the former methods mainly model drug-target interactions using features of small molecules, such as molecular fingerprints and pharmacophores, and the latter methods often rely on molecular docking to unveil potential interactions between small molecules and proteins. Both of these methods rely on the similarity assumption: “similar molecules target similar proteins or *vice versa*” (Sydow et al., 2019). However, this molecular similarity assumption does not always hold, e.g., structurally similar molecules can display different activities, such as the frequently observed activity cliffs (Bajorath, 2014). Moreover, ligand-based methods tend to exhibit decreased generalizability for new scaffold molecules that are not similar to any known drugs, and structure-based methods are limited by the lack of protein structures, inaccurate scoring functions, and a long computation time (Svensson et al., 2012).

The rapid accumulation of transcriptional profiling data provides a new perspective for computational target prediction. For example, the Library of Integrated Network-Based Cellular Signatures (LINCS) L1000 dataset (Subramanian et al., 2017) is a comprehensive resource of gene expression changes observed in human cell lines perturbed with small molecules and genetic constructs. Several computational methods that involve the exploration of differential expression patterns have been proposed (Bernardo et al., 2005; Lamb et al., 2006; Iorio et al., 2010; Chua and Roth, 2011; Woo et al., 2015; Filzen et al., 2017; Noh et al., 2018; Xie et al., 2018; Xu et al., 2018; Madhukar et al., 2019; Salviato et al., 2019), and the strategies used in these methods mainly include comparative analysis, network-based analysis, and machine learning-based analysis (Cereto-Massagué et al., 2015). The comparative analysis-based methods infer targets based on gene signature similarities (Lamb et al., 2006; Subramanian et al., 2017; Xu et al., 2018). An example is Connectivity Map (CMap), which assigns the target or mechanism of action (MOA) information of the most similar reference chemical/genetic perturbations to the new molecule by querying its gene expression signature against the reference L1000 library (Subramanian et al., 2017). The network-based approach systematically integrates gene expression profiles with cellular networks (Gardner et al., 2003; Cosgrove et al., 2008; Woo et al., 2015; Noh and Gunawan, 2016; Noh et al., 2018; Wang et al., 2020). For example, the mode-of-action by network identification (MNI) algorithm applies the network dynamics model learning from chemical perturbations and knockdown (KD) genetic perturbation to infer the drug targets ( Bernardo et al., 2005). ProTINA applies a dynamic model to infer drug targets from differential gene expression profiles by creating a cell type-specific protein-gene regulatory network and provides improved prediction results compared with similar methods (Noh et al., 2018). Different machine learning algorithms have also been used in mining transcription profile data, which have formal standardized statistical framework and optimization criteria and may show generalization capability. Pabon et al. implemented a random forest (RF) model to explore the correlations between compound-induced signatures (CP-signatures) and gene KD-induced signatures (KD-signatures) from CMap and predict drug targets (Pabon et al., 2018). Their study and that conducted by Liang et al. (2019) revealed that the comparison of the differential expression patterns induced by chemical perturbation with those induced by genetic perturbation might shed light on potential information on the targets of a compound. Because these gene expression profile-based methods go beyond relying on the structural similarity between molecules, they are more suitable for discovering the targets of molecules with novel scaffolds. For these machine learning models, a central question is how to incorporate information about biological graph such as protein-protein interaction networks. Conventional machine learning approaches often rely on summary graph statistics or carefully engineered features to measure local neighbourhood structures, which do not systematically consider the relationship among the nodes in biological networks (Hamilton et al., 2017). In addition, there are many other influencing factors, such as the effects of compound concentrations, the cellular background, and differences in the time scales between compounds and shRNAs, making the modelling more complicated. As a result, even if chemical and genetic perturbations interfere with the same target, the correlation between their gene signatures calculated using traditional methods might be very low because it is difficult to uncover the potential relevance of the gene signatures in biological networks under different conditions. To address this challenge, we propose a new graph convolution network (GCN) model, SSGCN. A trainable SSGCN was employed to integrate protein-protein interaction (PPI) information with raw signatures to derive graphical embeddings, and the results were then used to calculate the correlation between molecule-induced and KD-induced signatures. By concatenating the correlation results with the experimental CP time (the time from compound perturbation to measurement), dosages, cell lines, and KD time (time from KD perturbation to measurement), our model can predict drug targets across durations and dosages. Moreover, both external validations with LINCS phase II data and subsequently validated experimental findings demonstrate the usefulness of SSGCN in drug target identification and drug repositioning.

## Results

### Spectral-based GCN for learning the network perturbation similarities

To capture the drug-target interactions and thus identify drug targets, we propose a SSGCN model that learns the undiscovered correlations between CP-signatures and the corresponding KD-signatures at the network level.

#### Overall architecture of the model

**Figure 1:**
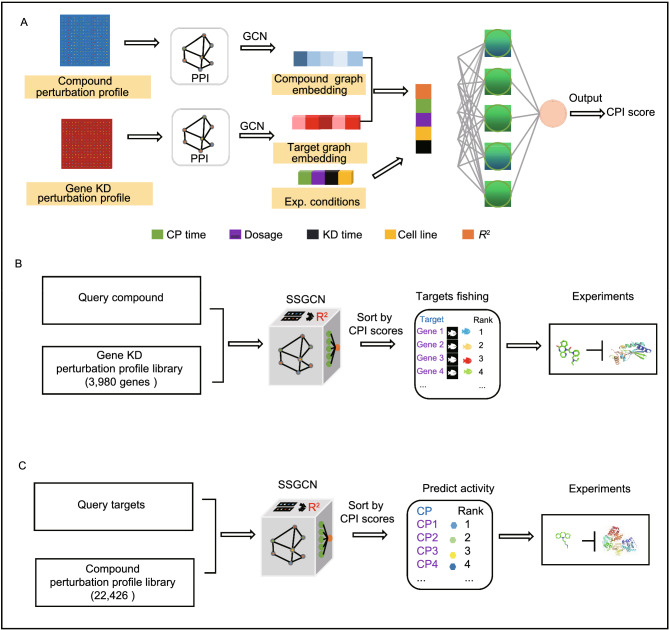
Target prediction using the SSGCN model. (A) Architecture of the SSGCN. Compound graph embedding is obtained by a spectral-based graph convolutional network (GCN) to integrate the protein-protein interaction (PPI) network topological structure information and compound perturbation profile. Target graph embedding is obtained by another GCN to integrate PPI and gene knockdown perturbation profile. The correlation coefficient Pearson *R*^2^ is calculated between the compound graph embedding and target graph embedding. The CP time is the duration of compound (CP) treatment and the KD time is the duration of gene knockdown (KD) perturbation. CPI score is the classification probability of whether the compound interacts with the protein. (B) Pipeline of the target inference using the SSGCN model. (C) Pipeline of identifying the novel active compound using the SSGCN model

The key idea of our target prediction model was to capture the correlations between chemical and genetic perturbation-induced gene expression in a more systematic manner. Based on this notion, targets of a compound can be predicted by comparing the corresponding perturbed gene expression profiles with a large number of KD-induced gene expression profiles that are publicly available. To learn potentially relevant information, as shown in Fig. 1A, two spectral-based GCNs were built: one for compound perturbation analyses, and one for gene perturbation analyses. This new architecture of the SSGCN model can also be divided into three main modules: the input module, the feature extraction module and the classification module. (1) The PPI network and differential gene expression profiles were the input of the first module. To unify information on the topology of the PPI network and the differential gene expression profiles, a property graph called a “gene signature graph” was constructed. Each node in the property graph represents a protein, and the property of each node was the corresponding differential gene expression value. Any two nodes are connected by an edge if two proteins can interact with each other. To represent compounds and targets, two gene signature graphs were constructed using compound and gene perturbation data. (2) In the feature extraction module, the spectral-based GCN was used for graph embedding to integrate the PPI network topological structure information and differential gene expression profiles. Graph embedding provides a compressed representation of the gene signature graph. To obtain graph embeddings of the compounds and targets, two parallel GCNs were established for feature extraction. Because vector operations are more efficient than operations on graphs, after the gene signature graphs were transformed into graph embeddings, a simple linear regression layer could be used to characterize the degree of correlation between these two graph embeddings of compounds and targets. Gene expression profiles are also related to cell types, durations, and compound dosages (Musa et al., 2018). Therefore, correlation values terms of Pearson *R*^2^ concatenated with the experimental meta-data (cell types, durations, and compound dosages) were fed into the classification module. (3) The classification module was composed of a fully connected hidden layer for extracting input features and an output layer for binary classification. The softmax function was applied in the output layer to compute the probabilities of whether the compounds show activity towards the potential targets (CPI scores). A label of 1 was assigned to a compound-protein pair if the compounds interacted with the corresponding protein, and a label of 0 was assigned to the opposite case.

The SSGCN model was implemented in the TensorFlow framework (version TensorFlow-GPU 1.14.0) in Python 3.7.

#### Target prediction with the SSGCN model

As shown in Fig. 1B, for a given compound C, the pipeline of predicting targets using the trained SSGCN model is as follows: (1) Obtain the compound perturbation gene profile on any of the eight cell lines, and extract the 978 landmark genes defined by the LINCS consortium (see METHODS for more details). In addition to L1000 assay, any cell-level transcriptomic profiling methods such as commercial gene expression microarrays or RNA sequencing (RNA-Seq) that could provide such information will be also applicable. We provided an “RNA-Seq application protocol” (a practical example included) in the Supplementary Information. (2) Feed the CP-signature and an existing KD-signature representing the gene perturbation profile of target **T** and their related experimental conditions, i.e., CP time, dosage, KD time, and cell line, to the trained SSGCN model for calculation of the CPI score of compound **C** and target **T**. (3) Repeat step 2 for the reference library of 179,361 KD-perturbation profiles. (4) Sort the potential targets by descending the mean CPI score of KD-perturbation profiles of the same target under different conditions. The top ranked targets are considered to be more likely to interact with compound **C**. Similarly, for a given Target **T** of interest, the pipeline can be reversely used to identify active compounds by screening the reference library of 22,426 CP-perturbation profiles (Fig. 1C).

### Optimization and internal test of the model using LINCS phase I data

**Figure 2:**
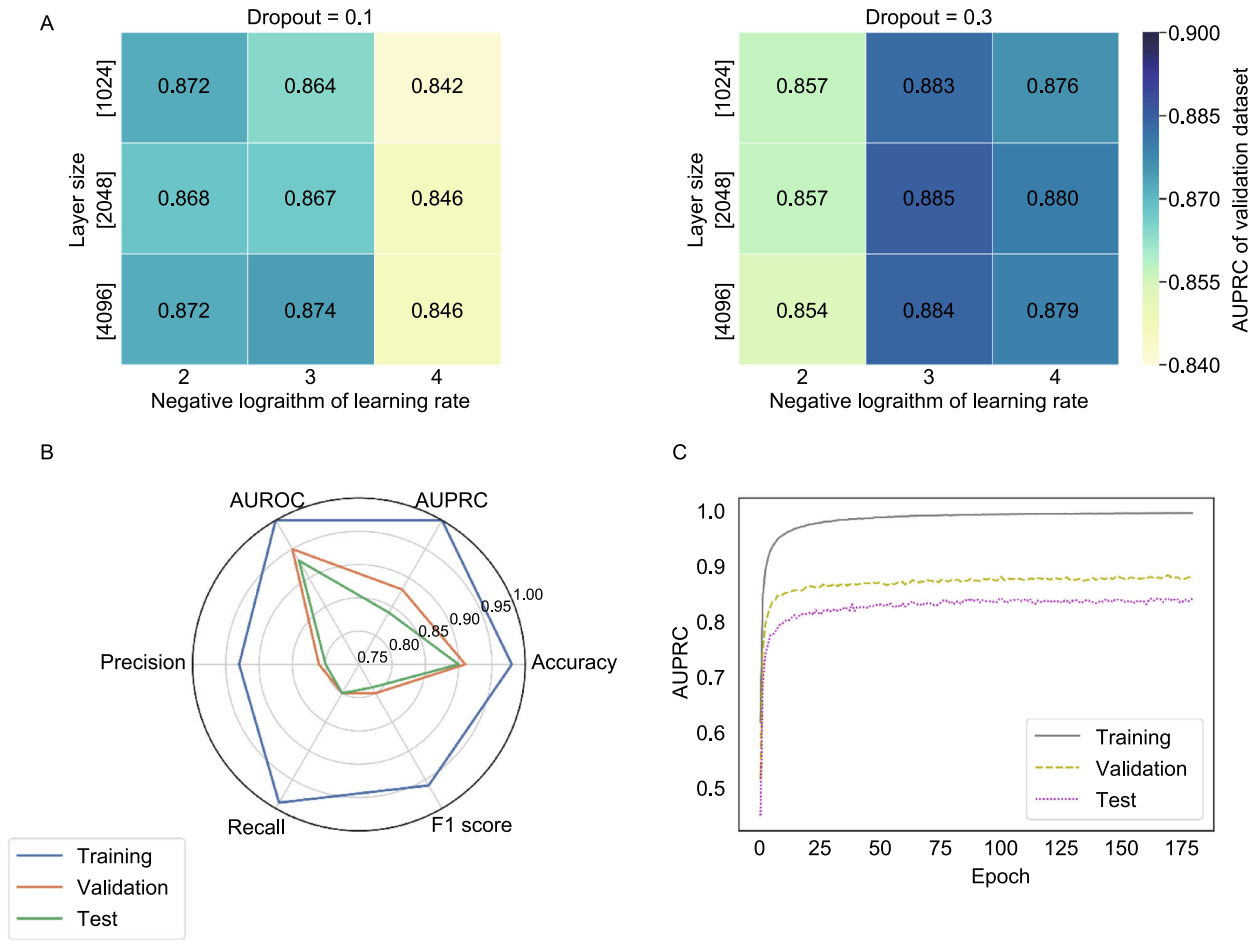
Heat maps for hyperparameters search. (A) The colormap reflects the magnitude of AUPRC (the area under precision recall curve) value on the validation dataset. The detailed description of the model evaluation metric can be found in the METHODS section of the article (Table 3). (B) Model performance shown in radar chart with six evaluation metric and (C) AUPRC-epoch curves. The “epoch” means an entire dataset is passed through a neural network once

The detailed process of data preprocessing can be found in the article METHODS section of the article. In general, the internal data set (training set, validation set, test set) and external test set are essential for modeling. Since the SSGCN model is sensitive to the combination of hyperparameters, hyperparameter search is important for model optimization. To optimize the model, as shown in Fig. 2A, different combinations of hyperparameters were evaluated with the validation dataset through grid searching. Because the number of negative samples was larger than that of positive samples (3:1), both the area under the precision-recall curve (AUPRC) and F1-score are more suitable for evaluating the classification performance of the model. As summarized in Fig. 2A, the final model showed the best performance on the validation set with a learning rate of 10^−3^, a layer size of 2,048, and a dropout of 0.3. As shown in Fig. 2B and 2C, the model has the best performance with an AUPRC of 0.84 and an F1 score of 0.79 on the test dataset when the epoch is 169.

### External test and model comparison using LINCS phase I data

#### Model performance and analysis using the external test set in LINCS phase I data

Although the model exhibited satisfactory results with the internal test dataset, we were more interested in its generalization ability for real-world target prediction tasks. Based on both the direct and indirect similarities between the chemical and KD perturbation signatures of cells, Pabon et al. applied an RF classification model to predict drug targets and constructed a dataset of 123 compounds and 79 targets, which could be considered a benchmark test for target prediction based on transcriptional profiles. To facilitate comparison, we used the same performance metric, top N accuracy, to evaluate the performance of our model. This metric reflects the proportion of tested compounds whose any true target can be correctly predicted among the top ranked N targets, and in this study, N values of 100 and 30 were evaluated. This is a non-stringent but well-accepted performance metric in the field of target inference. For example, a top 30 value close to 0.7 means that for a set 100 of test compounds, there are about 70 compounds whose real targets can be correctly ranked within the top 30 inferred targets list. The prediction results of the random forest model reported by Pabon et al. were directly used for model comparison. In addition, we also retrained the random forest model with our dataset. For further comparison, CMap was also implemented as a baseline model. For each compound in the external dataset, its top and bottom ranked 150 differentially expressed genes were used as the signature to query all the compounds in the LINCS phase I training data based on the CMap score. The value of the CMap score ranged from −100 to 100, where a large and positive value indicates that a reference compound could induce a signature similar to that induced by the query compound. Accordingly, all the known targets of the retrieved reference compounds with higher CMap scores were collected, and the top ranked 100 and 30 targets were assigned to the query compound as its candidate targets for calculating the top 100 and 30 accuracy values, respectively. Moreover, the network-based analytical method ProTINA was also benchmarked. Following the steps used in a previous study (Noh et al., 2018) and the provided code (https://github.com/CABSEL/ProTINA), the protein targets of the compound were ranked in descending order based on the magnitudes of the protein scores provided by ProTINA. It should be noted that different methods have different predicable target coverages. For SSGCN and the method reported by Pabon et al., the number of predicable targets corresponds to the number of different genes with available knockdown profiles in given cell lines. For CMap, the number is restricted to compound target-encoding genes. Among these methods, ProTINA covers more predicable targets because any genes with gene expression values can be considered potential targets. Finally, we reported the performance for a random prediction to indicate how these models are better than blind guessing.

**Figure 3:**
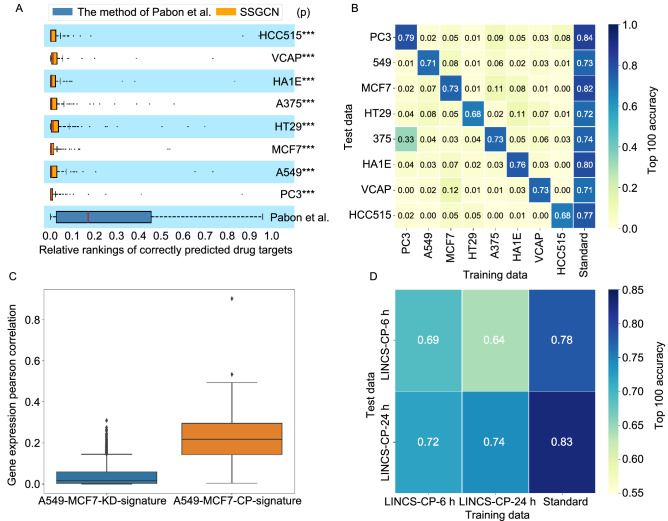
Model comparison and analysis. (A) Performance of the SSGCN models tested on different cell lines compared with that of the model developed by Pabon et al. (B) Effects of the cell lines on target prediction performance. The standard method is the SSGCN model trained on the KD profiles of all 8 cell lines. (C) The correlation between the KD signatures of A549 and MCF7 cells is significantly lower than that between the CP-signatures of these two cell lines. (D) Effects of the compound treatment time on target prediction performance

For a fair comparison, the gene expression profiles of these 123 compounds were excluded from the training dataset to avoid any potential information leakage. The remaining data were then used to train our model and predict targets for these 123 compounds according to the pipeline shown in Fig. 3. As shown in Table 1, the top 100 accuracy values of the model in eight cell lines were higher than 0.7, and the model tested on the PC3 cell line showed the best prediction performance. The relative ranks of the true targets were computed across eight cell lines. As shown in Fig. 3A and Table 1, our prediction accuracies on different cell lines were higher than those reported by Pabon et al. (***, *P* < 1 × 10^−10^), CMap (***, *P* < 1 × 10^−10^), ProTINA (***, *P* < 1 × 10^−10^), and random prediction (***, *P* < 1 × 10^−10^). It should be noted that retraining the RF model of Pabon et al. with our training set did not yield significant improvement in prediction, suggesting that the higher accuracy of SSGCN cannot be simply attributed to the introduction of more training data.

**Table 1:** Target prediction performance on the external test set in 8 cell lines

Methods	Number of compounds	Top 100 accuracy	Top 30 accuracy
SSGCN (PC3)	123	**0.84**	**0.71**
SSGCN (A549)	123	0.73	0.59
SSGCN (MCF7)	117	0.82	0.64
SSGCN (HT29)	123	0.72	0.46
SSGCN (A375)	122	0.74	0.58
SSGCN (HA1E)	123	0.80	0.63
SSGCN (VCAP)	120	0.71	0.43
SSGCN (HCC515)	111	0.77	0.63
RF (Pabon et al.)	123	0.26	0.14
RF (Using our training dataset)	123	0.27	0.17
CMap (PC3)	123	0.15	0.024
ProTINA (PC3)	120	0.033 (0.058)*	0.017 (0.033)*
Random prediction	123	0.02	0.008

^*^Because many more genes can be considered by ProTINA, the top 255 and 77 accuracy values, which denote the accuracy values at the same ratio of top 100 and 30 ranked targets, respectively, are also provided in parentheses for reference (255 = 100/3,980 ×10,174, 77 = 30/3,980 × 10,174). The bold means the best model

To analyse the effects of the cell lines on the prediction performance, the datasets were split according to their cell lines (PC3, A549, MCF7, HT29, A375, HA1E, VCAP and HCC515). Eight individual submodels were constructed for each cell line and then separately tested on the external test dataset. As shown in Fig. 3B, these submodels could not make transferable predictions across cell lines, with the exception of the submodel trained with the transcriptional data of PC3, which showed only moderate prediction capability (Top 100 accuracy = 0.33) on A375. The limitation of these submodels can be attributed to the poor correlation between the KD-signatures among different cell lines when interfering with the same gene. As revealed in the original study (Subramanian et al., 2017; Pabon et al., 2019), the similarity between shRNAs targeting the same gene is only slightly greater than random. Such similarity is even lower than that of signatures obtained after interfering with the same compound. Taking A549 and MCF7 as an example (Fig. 3C), the correlation of the KD signatures between these two cell lines was significantly lower than that of the CP-signatures. As shown in Fig. 3B, the standard method is the SSGCN model trained on the KD profiles of all 8 cell lines, and it shows good prediction performance on any of them. This result suggests that the application domain of the model can be expanded by further incorporating more data from different cell lines. Similarly, to analyse the effects of the CP time on the target prediction, two individual submodels for different time scales (6 h and 24 h) were built and tested. As shown in Fig. 3D, the models built from the LINCS-CP-6h dataset achieved a top 100 accuracy of 0.72 with the LINCS-CP-24 h test dataset, and those built from the LINCS-CP-24 h dataset achieved a top 100 accuracy of 0.64 with the LINCS-CP-6 h test dataset. These results showed that the model could make transferable predictions across CP times. In this study, the effects of the KD time on the target prediction were not analysed because most available KD-signatures were profiled at the same time (96 h, shown in Table S1).

#### The SSGCN model reveals a “deep correlation” between signatures

**Figure 4:**
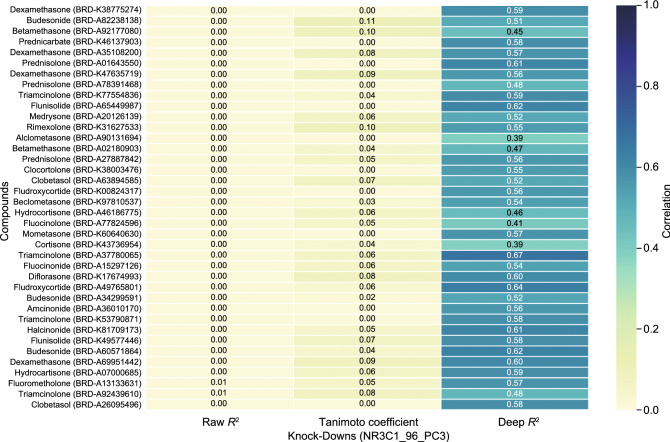
Correlation analysis of gene expression profiles. The raw *R*^2^, KEGG Tanimoto coefficient and deep *R*^2^ were used to represent the correlations of the raw gene expression values, KEGG pathway level features and graph embedding, respectively. NR3C1_96_PC3 means the gene NR3C1 knockdown profiles was selected with a duration of 96 h in the PC3 cell line

It is of interest to investigate whether our SSGCN model could help reveal the “deep correlation” that cannot be revealed by conventional normalization and scoring. Intriguingly, the external test set contains gene expression profiles of 38 different NR3C1 antagonists and thus constitutes an ideal subset for comparing expression profiles after different chemical and genetic interferences on the same target. Using this subset, the target NR3C1 of 11 ligands was identified among the top 100 candidate targets by the method developed by Pabon et al. In comparison, for all these 38 ligands, NR3C1 can be successfully predicted within the top 100 targets by our SSGCN model. As shown in Fig. 4, raw *R*^2^ and KEGG Tanimoto coefficient represent two conventional correlation scoring methods for comparing gene expression values or KEGG pathway level features. No significant correlation was found between the chemical and shRNA-induced gene expression profiles using these two methods. In contrast, the correlations calculated by comparing graph embeddings from the PPI network and differential gene expression profiles, termed deep *R*^2^, were markedly higher. These results highlight that our SSGCN model was able to determine the “deep correlation” between gene expression profiles upon heterogeneous drug treatments and explain why our model showed a markedly improved prediction performance in inferring targets based on transcriptional data.

### Model verification using LINCS phase II data

To further evaluate the generalization capability of the model in such a setting, LINCS phase II data were collected for stricter “time-split” testing (Sheridan, 2013). This dataset provides a more realistic prospective prediction setting in which the test data were generated later than the data used for modelling. After removing the overlapping compounds in the LINCS phase 1 data, the external test dataset includes 250 compounds and 488 targets. The trained model was employed to predict the targets of these compounds based on the target prediction pipeline shown in Fig. 1. For comparison, a baseline model, CMap, was again implemented.

**Figure 5:**
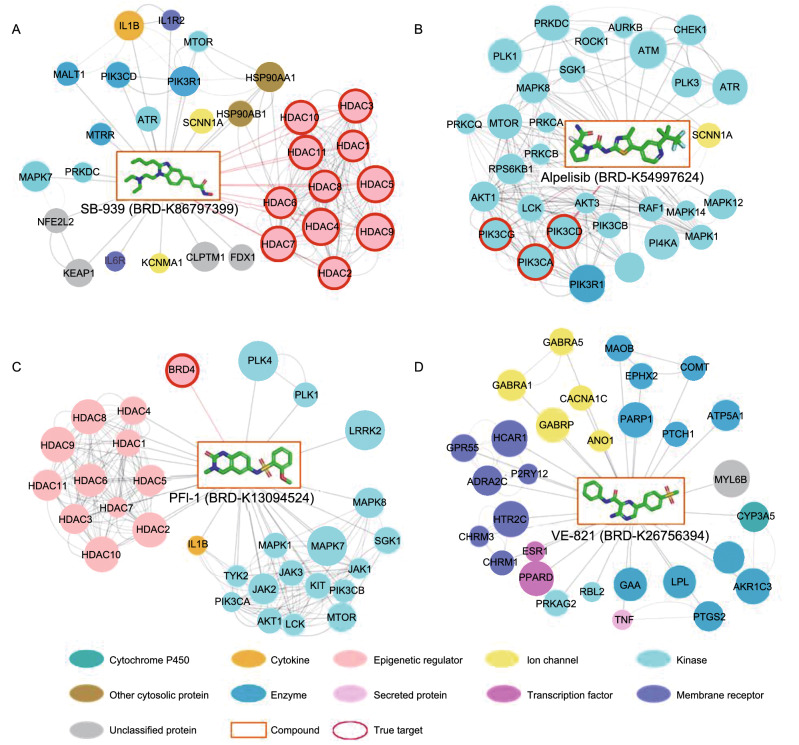
Examples of predicted targets (top 30) using the LINCS phase II data in PC3 cell lines. The following compounds were used for target prediction: (A) SB-939, (B) alpelisib, (C) PFI-1 and (D) VE-821. The nodes in rectangles represent compounds, and the nodes in circles represent the predicted targets. Predicted targets with a higher rank are indicated by a larger circle size. The corresponding true targets are indicated by red borders. The links between predicted targets denote protein-protein interactions that are curated from the STING database with a combined score greater than or equal to 800. Protein classification annotations come from ChEMBL database

The time-split validation represents a more rigorous estimate of the model performance. As summarized in Table 2, the top 100 accuracy values of the SSGCN on the time-split external test set ranged from 0.51 to 0.66 in six cell lines. Although the accuracy declined slightly compared with the previous internal test with phase I data, it might be caused by different coverages of the target space (Fig. S1) and batch effects such as temperature, wetness and different laboratory technicians (Leek et al., 2010; Subramanian et al., 2017), the overall results of the SSGCN model are still highly reasonable. In comparison, the baseline model using the CMap score for drug target prediction only yielded accuracy values lower than 0.31. We further performed a literature search for the discovered targets of these external test compounds. For example, MAPK14 was ranked at the 26th position of the potential targets for saracatinib, and we searched European patents and found that the *K*_d_ value of saracatinib for MAPK14 is 0.332 μmol/L. Similarly, MAPK1 was ranked at the 29th position among the potential targets of adenosine (Fedorov et al., 2007). This literature evidence further demonstrated the strong generalization capability of the SSGCN model for drug target prediction. For better visualization, a few external test compounds and their interaction network with the top 30 targets predicted by SSGCN are presented in Fig. 5 (more details are provided in Table S2). For example, the compound SB-939 is a potent pan-histone deacetylase (HDAC) inhibitor that inhibits class I, IIA, IIB and IV HDACs (HDAC1-11) (Novotny-Diermayr et al., 2010). As shown in Fig. 5A, the top ranked 11 targets for this compound were all HDACs, which are in accordance with the interacting targets reported previously. HDACs are the relatively easily predictable targets for transcription-only based target prediction methods, like CMap (Liu et al., 2018). Alpelisib is an oral α-specific PI3K kinase inhibitor that has shown efficacy in targeting PIK3CA-mutated cancer (André et al., 2019), and its combination with fulvestrant has recently been approved by the US Food and Drug Administration for the treatment of metastatic or otherwise advanced breast cancer. Interestingly, as shown in Fig.5B, the top ranked 30 targets of alpelisib are all types of different kinases, and PIK3CA can be successfully identified among the top three candidates. As a selective bromodomain-containing protein (BET) inhibitor, PFI-1 reportedly interacts with BRD4 with an IC_50_ of 0.22 μmol/L (Fish et al., 2012). As shown in Fig. 5C, BRD4 was ranked third in the list of candidate targets. Moreover, our model predicted that PFI-1 might show cross-activity with a range of kinases. Because an increasing number of studies have shown that BRD4/BET inhibitors and kinase inhibitors might act synergistically in a range of cancer types (Sun et al., 2015), the predicted off-target interactions with kinases might provide clues and starting points for further study of related dual functional inhibitors (Timme et al., 2020). In some cases, the predictions were unsuccessful, e.g., ATM and RAD3-related (ATR) kinase is a reported target of VE-821, but this target was ranked at the 1594th position. As shown in Fig. 5D, the top 30 ranked targets identified by SSGCN cover a wide range of protein categories, including kinases, GPCRs and ion channels. Because compounds with smaller molecular weights might show promiscuity across different target families, we cannot rule out the possibility that VE-821 interacts with the predicted targets, but none of these interactions are supported by reported experimental evidence. This example also suggested that the candidate target list should be refined through further experimental verification and combination with other complementary methods, such as structure-based or similarity-based approaches. Moreover, we studied the relationship between protein family and prediction performance of the SSGCN model (Fig. S2). Among the 100 targets giving the best performing predictions, we may find that a wide range of different types of protein targets are included, not only epigenetic regulators or kinases that may induce strong transcriptional signatures, but also other enzymes, ion channels and membrane receptors. These results suggest that our model indeed learns the ability of target inference, but not simply remembers some eminent transcriptional features. Overall, as indicated in Table 2 and Fig. 5, it can be concluded that the SSGCN model shows strong generalization ability for inferring targets of previously unevaluated compounds and provides insights on cell-level transcriptomic responses to chemical intervention and related polypharmacological effects.

**Table 2:** Target prediction performance on the LINCS phase II data

Cell lines	Number of compounds	Top 100 accuracy (SSGCN)	Top 30 accuracy (SSGCN)	Top 100 accuracy (CMap)	Top 30 accuracy (CMap)
PC3	249	**0.53**	0.30	0.29	0.12
A549	41	**0.66**	0.51	0.31	0.20
MCF7	240	**0.53**	0.30	0.24	0.10
A375	245	**0.51**	0.31	0.30	0.15
HA1E	238	**0.56**	0.34	0.27	0.13
HCC515	39	**0.65**	0.46	0.15	0.05

The bold means the best model

### Compound-centric prediction of Cyclophilin A as a novel target for nelfinavir

Nelfinavir (NFV) is a potent protease inhibitor that has been widely used for many years for the treatment of human immunodeficiency virus type 1 (HIV-1) infection. Recently, there is a rapidly expanding literature on the *in vitro* anti-SARS-CoV-2 activity of NFV, which includes NFV significantly inhibited SARS-CoV-2 replication in Vero E6 cells (Arshad et al., 2020; Ianevski et al., 2020; Ohashi et al., 2020; Xu et al., 2020a, b; Yamamoto et al., 2020), *in silico* modeling showed NFV bound to SARS-CoV-2 main protease consistent with its inhibition of viral replication (Ohashi et al., 2020; Xu et al. 2020). Besides, another *in silico* modeling also suggested that NFV may bind inside the S trimer structure and thus inhibited SARS-CoV-2 spike-mediated cell fusion, suggesting that NFV may efficiently inhibit the spread of SARS-CoV-2 from cell-to-cell (Musarrat et al., 2020). A major underlying cause of COVID-19 patient mortality is a hyperinflammatory cytokine storm syndrome in severe/critically ill patients (Huang et al., 2020). NFV has been reported to significantly inhibit inflammatory cytokines *in vitro* (Equils et al., 2004; Wallet et al., 2012), and to reduce inflammatory cytokine in a cohort of pediatric HIV-1 patients for over 2 years of the therapy (Wallet et al., 2012), which may be possible to help alleviate the cytokine storm syndrome of COVID-19. However, the anti-viral and/or anti-cytokine-storm human targets of NFV have never been identified and reported in the literature. Thus, investigations of the potential anti-viral and/or anti-cytokine-storm human targets of NFV is considered to be a significant work.

Therefore, we experimentally verified compound-centric target inference pipeline (Fig. 1) by analyzing the gene expression profile of NFV perturbation and potential target protein-NFV direct binding. For the top 30 targets predicted for NFV via the compound-centric target inference pipeline, Calcineurin B, type II (CNBII, also known as PPP3R2), Cyclophilin A (CYPA, also known as PPIA) and Calcineurin A alpha (CNA1, also known as PPP3CA) were ranked 2th, 7th and 13th respectively and caught our attention. It has been reported that the outcome of COVID-19 in a cohort of patients undergoing treatment with calcineurin inhibitors is promising, mainly due to the immunosuppressive role for calcineurin inhibitors (Cavagna et al., 2020). CYPA has been reported to regulate viral infectivity (Braaten and Luban, 2001), and its inhibition could inhibit the replication of coronaviruses and the inflammatory cytokine expression and inflammation (Tanaka et al., 2013; Dawar et al., 2017). It’s well known that CYPA and calcineurin are the upstream regulators of nuclear factor of activated T cells (NF-AT) activity, inhibition of CYPA and/or calcineurin blocks the translocation of NF-AT from the cytosol into the nucleus, thus preventing the expression of interleukin-2 (IL-2) (Tanaka et al., 2013).

**Figure 6:**
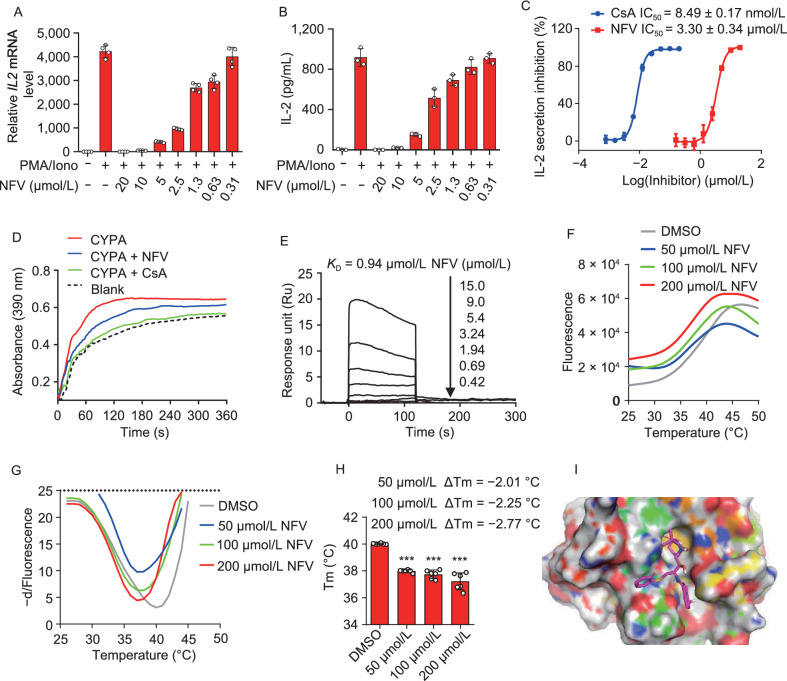
Compound-centric prediction of CYPA as a novel target for NFV. (A–C) NFV inhibited the transcription and secretion of IL-2 in a dose-dependent manner. Jurkat T cells were treated with different concentration of NFV or CsA for 2 h, following stimulation with PMA (100 nmol/L) and Ionomycin (10 μmol/L) for 24 h. After treatment, cells and culture supernatant were collected and subjected to RT-qPCR and ELISA. *IL2* mRNA levels were normalized to *ACTB* and fold induction was calculated relative to untreated cells, data showed pooled technical replicates from three independent experiments. (D) CYPA peptidyl-prolyl *cis-trans* isomerase (PPIase) activity was assessed using the α-chymotrypsin-coupled assay. Isomerization of the succinyl-AAPF-pNA peptide substrate was reflected by an increase in absorbance at 390 nm. The curves represent isomerization of this substrate at 4 °C over the course of 360 s in the absence of CYPA (Blank), or in the presence of 2 μmol/L CYPA, or in the presence of 2 μmol/L CYPA incubated with 10 μmol/L NFV or CsA. Data are representative of three independent experiments with similar results. (E) NFV bound to CYPA protein as shown by surface plasmon resonance measurements. Graphs of equilibrium response unit responses versus compound concentrations were plotted. (F–H) Thermostability of CYPA treated with 0, 50, 100, 200 μmol/L NFV. The thermal stability of CYPA was quantified by the ΔT_m_ in pooled technical replicates from at least three independent experiments. Data are represented as mean ± SD (*n* = 6), ***, *P* < 0.001; by 2-tailed, unpaired *t*-test. (I) The putative binding mode of NFV (stick) to human CYPA (surface, 2X2C). Error bars represent SD around the mean (A–C, H)

Given the possibility that NFV is a potential CYPA or calcineurin inhibitor, we firstly measured the transcription and secretion of IL-2 in Jurkat T cells upon phorbol 12-myristate 13-acetate (PMA) and ionomycin stimulation. The results showed that NFV inhibited transcription of *IL2* in a dose-dependent manner (Fig. 6A). Similarly, NFV also inhibited the secretion of IL-2 in a dose-dependent manner and IC_50_ was 3.30 ± 0.34 μmol/L (the inhibition rate was almost 100% at 20 μmol/L), which was inferior than IC_50_ of cyclosporine A (CsA) (8.49 ± 0.17 nmol/L) (Fig. 6B and 6C), a well-known immunosuppressive drug that is the main inhibitor of CYPA (Tanaka et al., 2013). These results inspired us to conduct further experiments to confirm the possibility that NFV is a potential CYPA or calcineurin inhibitor. We then evaluated the potential of NFV to inhibit the calcineurin phosphatase activity using the RII phosphopeptide as substrate, and the results showed that NFV had no obvious effect on calcineurin phosphatase activity (Fig. S3). Therefore, we immediately performed chymotrypsin-coupled CYPA peptidyl-prolyl *cis-trans* isomerase (PPIase) activity assay to test whether NFV can affect the PPIase activity of CYPA. The results showed that NFV exhibited significant inhibition of CYPA PPIase activity, while the role was weaker than CsA (Fig. 6D). To determine whether NFV directly bind to CYPA and inhibit its activity, we examined the direct binding of NFV to purified CYPA in vitro using surface plasmon resonance technology. As shown in Fig. 6E, the binding curve of NFV showed a fast-on, fast-off kinetic pattern in dose-dependent manner with a *K*_D_ of 0.94 μmol/L. Furthermore, we measured the thermal stability of purified CYPA in the presence of NFV. Protein thermal shift assay showed that NFV destabilized CYPA conformation and decreased the melting temperature (Tm) in a dose-dependent manner (Fig. 6F–H), suggesting direct NFV-CYPA binding. Although the ligand induced protein destabilization is not typical, it has been frequently observed in the specific binding of inhibitors to enzymes (Zhao et al., 2015; Pacold et al., 2016). Here, we argue that NFV may destabilize the native conformation of CYPA upon binding preferentially to its less populated conformational state (Cimmperman et al., 2008; Kabir et al., 2016), but the exact mechanism is not clear and falls outside of the scope of the current study. To gain the binding mode between NFV and CYPA, we docked the NFV to the structure of CYPA (PDB ID: 2X2C). The docking result showed that the NFV occupied the catalytic pocket at the binding site (Fig. 6I), which may explain how NFV affects the PPIase activity of CYPA. Taken together, these results showed that NFV directly binds to CYPA and inhibits its activity, and CYPA is a novel target for NFV. It has been demonstrated that low concentration of IL-2 effectively prevents excessive inflammation in a wide range of preclinical models of inflammatory diseases, including beryllium-induced lung inflammation, by maintaining activity and survival of T regulatory cells (Treg) that play a crucial role in the control of immune responses, in part by inhibiting overactive inflammation, while high concentration of IL-2 has an opposite effect inducing cytokine storm (Hirakawa et al., 2016; Abbas et al., 2018; Xu et al., 2019). COVID-19 disease severity is associated with high plasma level of IL-2, which may be considered therapeutic targets for COVID-19 to combat hyperinflammatory responses and cytokine storms (Behm et al., 2020; Huang et al., 2020). The efficacy of low dose IL-2 in improving clinical course and oxygenation parameters in COVID-19 patient is now in clinical phase II trials (NCT04357444). Based on these effects of NFV on CYPA activity and IL-2 production, further research of NFV's effect in human COVID-19 patients is warranted.

### Target-centric prediction of methotrexate as a novel ENPP1 inhibitor

Stimulator of interferon genes (STING) is an endogenous sensor of cGAMP, which is synthesized by cyclic GMP-AMP synthase (cGAS) following detection of cytoplasmic DNA. STING activation leads to interferon production and downstream innate and adaptive immune responses (Corrales et al., 2015). Ectonucleotide pyrophosphatase/phosphodiesterase-1 (ENPP1) is the phosphodiesterase that negatively regulates STING by hydrolyzing cGAMP (Li et al., 2014). It is pivotal and significant to develop ENPP1 inhibitor for cancer immune therapy.

**Figure 7:**
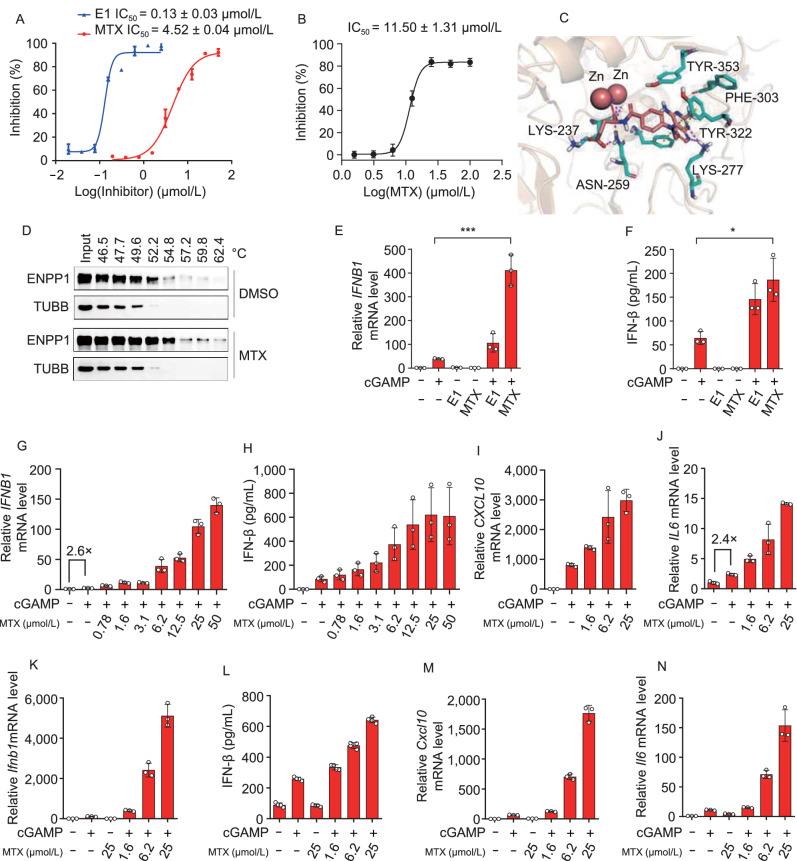
Target-centric prediction of MTX as a novel ENPP1 inhibitor. (A and B) Inhibition of MTX and E1 on hydrolysis of p-Nph-5’-TMP (A) or ATP (B) by ENPP1 *in vitro*. (C) The *in silico* simulation analysis of the binding site of the ENPP1 (cyan, 4GTW) with MTX (violet). (D) Representative immunoblot for the effect of MTX on thermal stability of ENPP1 protein in cellular thermal shift assay. 293T cell lysates with or without MTX (50 μmol/L) treatment were incubated at different temperatures, then ENPP1 turnover was monitored by Western blot. (E and F) MTX and E1 increased the transcription (E) and secretion (F) of IFN-β in cGAMP treated THP-1-derived macrophages. THP-1-derived macrophages were treated with MTX (20 μmol/L) or E1 (20 μmol/L), following stimulation with cGAMP (500 nmol/L) for 24 h, then cells and culture supernatant were collected and subject to RT-qPCR and ELISA. Data are represented as mean ± SD (*n* = 3). *, *P* < 0.05; ***, *P* < 0.001; by 2-tailed, unpaired *t*-test. (G–J) MTX increased the transcription of *IFNB1* (G), *CXCL10* (I), *IL6* (J) and secretion of IFN-β (H) in a dose-dependent manner in cGAMP treated THP-1-derived macrophages.THP-1-derived macrophages were treated with the indicated concentration of MTX, following stimulation with cGAMP (500 nmol/L) for 24 h, then cells and culture supernatant were collected and subjected to RT-qPCR and ELISA. (K–N) MTX increased the transcription of *Ifnb1* (K), *Cxcl10* (M), *Il6* (N) and secretion of IFN-β (l) in a dose-dependent manner in cGAMP treated RAW 264.7 cells. RAW 264.7 cells were treated with the indicated concentration of MTX, following stimulation with cGAMP (5 μmol/L) for 24 h, then cells and culture supernatant were collected and subjected to RT-qPCR and ELISA. All above data showed pooled technical replicates from three independent experiments. mRNA levels were normalized to *ACTB* and fold induction was calculated relative to untreated cells. Error bars represent SD around the mean (A, B, E–N)

As shown in Fig. S4, the pipeline of the target-centric prediction was applied to find the novel ENPP1 inhibitor. The reference library of 22,425 compound perturbation profiles in the PC3 cell line were screened, and those compounds with the CPI score greater than 0.5 were selected, leading to 190 compounds considered potentially active against ENPP1. Considering the complexity of biological networks, complementary approaches should be integrated to produce the most reliable target and mechanistic hypotheses (Schenone et al., 2013). In computational target inference, Pabon et al. have also demonstrated that molecular docking will reduce the false positives and further enrich predictions of model based on transcriptomics (Pabon et al., 2018, 2019). Therefore, we also incorporated the structural screening as an orthogonal approach in the pipeline, and we docked the 190 compounds to structures of ENPP1 (PDB ID: 4GTW) and selected the top ranked 7 available compounds for further experiment validation. We firstly evaluated the potential of these 7 compounds to inhibit the ENPP1 enzyme activity in vitro using thymidine 5’-monophosphate p-nitrophenyl ester (p-Nph-5’-TMP) as substrate, the results showed methotrexate (MTX) displayed promising inhibition activity (>50%) at the concentration of 10 μmol/L, which was identified as an ENPP1 inhibitor with IC_50_ of 4.52 ± 0.04 μmol/L (Figs. 7A and S5), while the effect was weaker than the reported ENPP1 positive inhibitor ENPP-1-IN-1 (E1) (Gallatin et al., 2019). Similar ENPP1 inhibition effect of MTX was observed using ATP as substrate by Liquid chromatography and tandem mass spectrometry (Fig. 7B). To gain the structural insight of the interaction between MTX and ENPP1, we docked MTX with mouse ENPP1 (PDB ID: 4GTW). As shown in Fig. 7C, hydrogen bonds were formed between N (1), N (8) atoms of pteridine ring and LYS-277, -NH2 (2) of pteridine group and PHE-303. In addition, pi-pi stacking interactions were formed between the pteridine ring and TYR-322, PHE-239. These interactions might lock pteridine moiety in the pocket tightly. Moreover, a salt bridge and another hydrogen bond were formed between the tail carboxyl groups and zinc ions, LYS-237, which might make the conformation of MTX more stable in the pocket. To further verify the interaction between MTX and ENPP1 protein, cellular thermal shift assay (CETSA) was performed. The thermostability of ENPP1 in 293T cell lysates with or without 50 μmol/L MTX was analyzed. As showed in representative western blot (Fig. 7D), the detected soluble ENPP1 protein exhibited a clear difference between being untreated and treated with MTX at denaturation temperatures ranging from 52 °C to 62 °C, indicating MTX directly bound to the ENPP1 protein. To assess the effect of ENPP1 inhibition by MTX, we detected representative STING-TBK1-IRF3 pathway downstream cytokines. As expected, MTX enhanced transcription and secretion of interferon beta (IFN-β) induced by 500 nmol/L cGAMP in THP-1-derived macrophages, while MTX alone administration didn’t (Fig. 7E and 7F), indicating the enhancement was due to inhibition of cGAMP hydrolysis. In same condition, MTX showed more effective activation than the reported ENPP1 positive inhibitor E1 (Fig. 7E and 7F). MTX enhanced transcription of *IFNB1* (Fig. 7G), *CXCL10* (Fig. 7I), *IL6* (Fig. 7J) and secretion of IFN-β (Fig. 7H) induced by cGAMP in THP-1-derived macrophages in a dose-dependent manner. However, MTX could not enhance the transcription of *IFNB1* induced by GSK3 (Fig. S6), another STING activator that does not have phosphodiester linkage (Ramanjulu et al., 2018). Besides, MTX didn’t show cytotoxicity at up to 100 μmol/L in THP-1-derived macrophages (Fig. S7). Similar STING pathway activation results were observed in RAW 264.7 cells (Fig. 7K–N). Taken together, MTX was identified as an ENPP1 inhibitor that promoted STING activation *in vitro*. By inhibiting dihydrofolate reductase, MTX was originally developed and continues to be used for the treatment of various types of cancer including breast cancer (Sramek et al., 2017). Radiation therapy, commonly used to treat cancer, was reported to increase cytosolic DNA and induce STING activation (Carozza et al., 2020). Our findings validated the SSGCN prediction that MTX can be repurposed toward ENPP1. Furthermore, MTX promoted STING pathway activation by inhibiting ENPP1 and provided clinical potential for combining MTX with radiation therapy for the treatment of breast cancer in which ENPP1 shows hyper-expression (Carozza et al., 2020).

## Discussion

The drug-induced perturbation of cells leads to complex molecular responses upon target binding, such as the feedback loop that changes the expression level of the target node or its upstream and downstream nodes. These drug-induced responses likely resemble those produced after silencing the target protein-coding gene, which provides a rationale for comparing the similarity between chemical- and shRNA-induced gene expression profiles for target prediction (Pabon et al., 2018). The encoding and denoising of a given experiment’s transcriptional consequences constitute a challenge. In this study, we proposed a new deep neural network model, the Siamese spectral-based graph convolutional network (SSGCN), to address this challenge.

The SSGCN model takes two differential gene expression networks (a chemical-induced network and a shRNA-induced network) as input and integrates heterogeneous experimental condition information to account for variances such as cell line-, dose- and time-dependent effects. By training using known compound-target interaction data, the model can automatically learn the hidden correlation between gene expression profiles, and this “deep” correlation was then used to query the reference library of 179,361 KD-perturbation profiles with the aim of identifying candidate target-coding genes. The pipeline improved target prediction performance on a benchmark test set. For more rigorous time-split validation using LINCS phase II data, the target prediction results obtained with our method achieved better performance compared with those achieved with the conventional CMap-based approach. Furthermore, to test the practical usefulness of the approach, we simulated two potential application scenarios and experimentally verified the prediction results. In the first case, a compound-centric target inference pipeline (Fig. 1B) was established to identify the potential host targets of nelfinavir (NFV). In the second case, the pipeline of a target-centric prediction was established to find novel small molecule inhibitors of ectonucleotide pyrophosphatase/phosphodiesterase 1 (ENPP1), by screening 22,425 compound perturbation profiles. Our experimental findings successfully validated that Cyclophilin A (CYPA) ranked 7th place is a novel target of NFV, and methotrexate (MTX) may promote STING pathway activation by inhibiting ENPP1. These two examples highlight our model as a useful tool to infer the interacting targets of active compounds, or reversely, to find novel inhibitors of a given target of interest. Moreover, we checked the similarity between the predicted and the known drug-target interaction pairs. The maximum chemical similarity between MTX and the known ENPP1 inhibitors is 0.23, and the maximum chemical similarity between NFV and the known CYPA inhibitors is 0.22; The highest homology between CYPA and known targets of NFV is 0.06773, and the highest homology between ENPP1 and known targets of MTX is 0.1008. These results indicate that our model is orthogonal to standard approaches based on chemical/protein similarities and can identify novel drug-target interactions, and clearly demonstrate the importance of SSGCN as an orthogonal approach to the conventional similarity based approaches. Overall, the SSGCN model allows in silico target inference based on transcriptional data and is of practical value for repurposing existing drugs or exploring the MOA of not-well-characterized bioactive compounds and natural products.

## Methods

### Materials and methods

#### Data collection

LINCS: The Library of Integrated Network-Based Cellular Signatures (LINCS) program, which is funded by the NIH, generates and catalogues the gene expression profiles of various cell lines exposed to a variety of perturbing agents in multiple experimental contexts. Both the LINCS phase I L1000 dataset (GSE92742, 2012–2015) and the LINCS phase II L1000 dataset (GSE70138, 2015–2020) were downloaded from the Gene Expression Omnibus (GEO) provided by the Broad Institute. These profiles were produced by a high-throughput gene expression assay called the L1000 assay, in which a set of 978 “landmark” genes. This reduced “landmark” gene set enabled the LINCS program to generate a million-scale transcriptional profile. For the sake of connectivity analysis and convenience, our analysis focused on the level 5 signature data (replicate-collapsed z-score vectors) and used only real measured expression values of the landmark genes. The Python library cmapPy (Enache et al., 2019) was used to access the level 5 signatures from GCTx files.

STRING: STRING (Szklarczyk et al., 2019) is a database compiled for PPIs from both known experimental findings and predicted results. The human PPI network from the STRING v11.0 database was downloaded.

#### Data preprocessing

**Figure 8:**
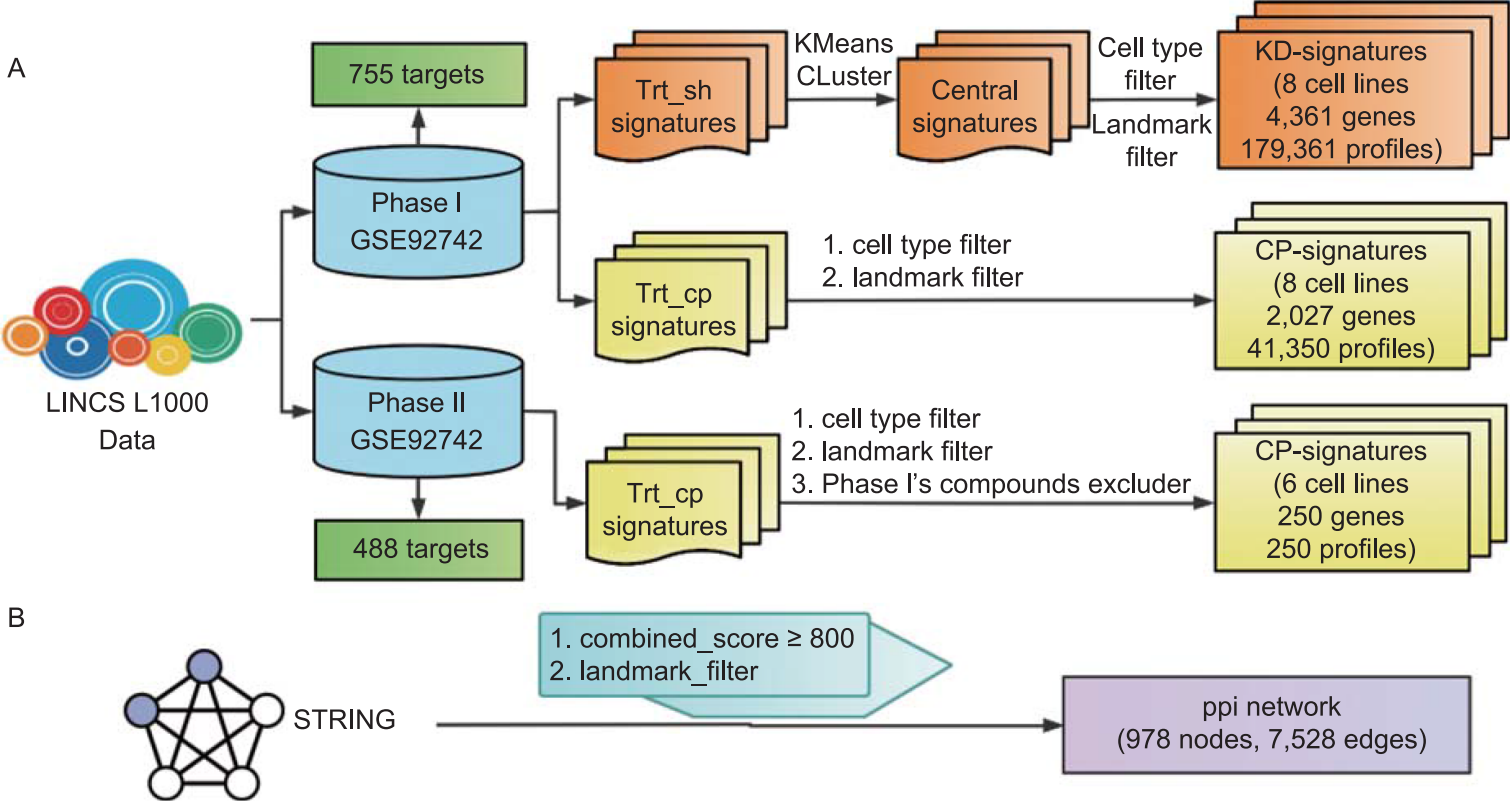
Pipeline of the data processing. (A) Processing pipeline for LINCS L1000 data. (B) Processing pipeline for STRING v11.0 PPI data. “trt_sh” and “trt_cp” are official tags that denote knock down treatment and compound treatment in LINCS dataset respectively. “cell type filter” filtered out other cell type data except those in eight cell lines (A375, A549, HA1E, HCC515, HT29, MCF7, PC3, and VCAP). “Landmark filter” filtered out other gene values in signatures except those in 978 “landmark” genes. The “combined score” is measure score offered by STRING database for the confidence of several types of evidence which support a protein-protein association

LINCS: The pipeline used for the preprocessing of the LINCS dataset is shown in Fig. 8A. (1) Profile signatures after perturbation with shRNAs (Phase I). shRNA experiments might exhibit off-target effects due to the “shared seed” sequence among shRNAs (Jackson et al., 2003; Subramanian et al., 2017). To gain an abundant set of robust KD signatures, we performed k-mean (k = 1) clustering of the “trt_sh” signatures separated by the cell lines and KD time and maintained the core signature, which is the central signature of the cluster, as a representation of the corresponding cluster (Xie et al., 2018). The core signatures across eight data-rich cell lines (A375, A549, HA1E, HCC515, HT29, MCF7, PC3, and VCAP) were filtered to obtain the corresponding 978 “landmark” vectors, which are 978 differential gene expression values defined by the LINCS consortium. These 978 vectors constituted the input of curated KD signatures. (2) Profile signatures after perturbation with compounds (phase I). The targets of the compounds were retrieved using the application programming interface (API) from the cloud platform (clue.io) provided by the Broad Institute. This retrieval resulted in 2,027 compounds with 755 targets. Consistent with the curated KD signatures, CP-signatures were curated by filtering “trt_cp” signatures out of the data-poor cell lines and non-landmark vectors. (3) Profile signatures after perturbation with compounds (phase II). We first filtered out those compounds contained in the phase I dataset and then retrieved the targets of the compounds from the aggregated ChEMBL bioactivity data on LINCS Data Portal through a representational state transfer API (Koleti et al., 2018). The targets with pKd, pKi or pIC_50_ values greater than or equal to 6.5 were treated as the “true” targets (Lenselink et al., 2017). The retrieval resulted in 250 compounds with 488 targets. The raw signatures of these 250 compounds across eight data-rich cell lines (A375, A549, HA1E, HCC515, HT29, MCF7, PC3, and VCAP) were then extracted from the LINCS phase II dataset. As mentioned above, only the 978 “landmark” vectors were retained. We preferred to select the samples with a dosage of 10 μmol/L and a duration of 24 h, and for the data without a dosage of 10 μmol/L or a duration of 24 h, the gene signature for the closest conditions is used as an alternative.

STRING: We only kept the nodes present in the “landmark” gene set and the PPI edges with a “combined score” greater than or equal to 800. Accordingly, the curated PPI network consists of 978 nodes and 7,528 edges (Fig. 8B).

#### Data sampling

The test set compiled by Pabon et al., which contained 123 FDA-approved drugs that had been profiled in different LINCS cell lines and whose known targets were among the genes knocked down in the same cells, was used for benchmarking. Moreover, another benchmark dataset was prepared based on 250 compounds from LINCS phase II. The test dataset compiled by Pabon et al. and the dataset from LINCS phase II are taken as two external datasets. After excluding CP-signatures in these two external datasets, the remaining data of the phase I of LINCS database is regarded as the internal dataset. The internal dataset was divided into three sets: training, validation, and test data set in the ratio of 8:1:1, by random splitting based on chemical structures. In different drug discovery projects, the proportion of active compounds may vary significantly but in most cases those inactives appear more often than actives. Here, for each compound three negative targets were generated for each positive target through a random cross combination of compounds and proteins. In addition, the performance of the model trained with different data proportions was discussed in Fig. S8.

### Definition of the spectral-based GCN

An undirected graph G with 978 nodes was applied to represent the landmark PPI network. Each node in graph G represents a protein, and each edge represents a specific PPI interaction. Neighbourhood information is included in the edges. Traditional convolutional neural network structures are unfit for convolution operations on this graph, which is a non-Euclidian structure. Based on the Fourier transform of the graph and convolution theorem, spectral-based convolution operations on the graph can be applied to capture the properties of the graph network (Bruna, 2014).

For a given graph $\mathrm{G}$, its Laplacian matrix $L$ can be defined as

1 $$L = D - A,$$

where $A$ is the adjacency matrix of graph $\mathrm{G}$ and $D$ is the degree matrix of graph $\mathrm{G}$. In graph theory, the symmetric normalized Laplacian is more often used due to its mathematical symmetry. The symmetric normalized Laplacian ${L}_{sys}$ can be defined as

2 $$L_{sys} = D^{ - 1/2} LD^{ - 1/2} .$$

Based on the classical Fourier transform, we redefined the Fourier transform of the feature function in the node as the inner product of the function and the corresponding eigenvectors of the Laplacian matrix:

3 $$\hat{f} = \left\langle {f,{ }v_{k} } \right\rangle ,$$

where $k$ is the node on the graph, $f$ is the feature function in node $k$, and ${v}_{k}$ is the eigenvector in the node of the Laplacian matrix. If spectral decomposition is performed on the Laplacian matrix, ${L}_{sys}$ can be expressed as

4 $${L}_{sys} = U \lambda {U}^{T}$$

$U$ is the orthogonal matrix of which the column vector is the eigenvector of the Laplacian matrix and $\lambda$ is the diagonal matrix in which the diagonal is composed of the eigenvalues. The Fourier transform of the feature function $f$ on the graph can then be rewritten as

5 $$\hat{f} = U^{T} f$$

Because $U$ is an orthogonal matrix, the inverse Fourier transform of function $f$ on the graph can be written as

6 $$f = U\hat{f}.$$

According to the convolution theorem in mathematics, a convolution procedure of two functions is the inverse Fourier transform of the product of their Fourier transforms. Defining $h$ as the convolution kernel, the convolution operation on the graph can be expressed as

7 $$\left( {f{*}h} \right)_{{graph{ }}} = U\left( {\left( {U^{T} h} \right)\left( {U^{T} f} \right)} \right).$$

For the convolution operation in the first layer of the GCN, the Fourier transform of $h$ is directly defined as the trainable diagonal matrix ω. Therefore, the convolution operation on the graph can be expressed as

8 $$\left( {f{*}h} \right)_{{graph{ }}} = U\omega U^{T} f.$$

After the above derivation, the final form of the single layer of the spectral-based GCN can be expressed as

9 $$H_{{n + 1{ }}} = \sigma \left( {U\omega U^{T} H_{n} } \right).$$

where $\sigma$ is the activation function of the layer, ${H}_{n}$ is the input features of layer ${n}_{th}$, and ${H}_{n+1}$ is the output of layer $({n+1)}_{th}$. According to the above definitions, the spectrum (eigenvalue) plays an important role in the convolution operation; thus, the GCN is called the spectral-based GCN. To effectively extract features and deeply learn from data, the multilayer perceptron can be connected to the graph convolution layer to increase the capacity of the model.

### Training protocol

The model was trained on the training set using the Adam optimizer (Kingma and Ba, 2014). The model was trained to minimize the cross entropy between the label and the prediction result as follows:

$$loss = - \frac{1}{n}\sum \left[ {y lnp + \left( {1 - y} \right)\ln \left( {1 - p} \right)} \right],$$

where $p$ refers to the prediction result and $y$ refers to the label. Early stopping was used to terminate the training process if the performance of the model on the validation dataset shows no further improvement in specified successive steps, which helps selection of the best epoch and avoid overfitting. The computational performance took 2–3 h to train the model (through 380 epochs and 24 s each) with a NVIDIA TITAN RTX graphics processing unit (GPU) on an Intel platform.

### Model evaluation metric

The predictive performance of the model on the test set was evaluated using six classification metrics: accuracy, precision, recall, F1 score, area under the receiver operating characteristic (ROC), and area under the precision-recall curve (PRC). TP is the number of true positives, TN is the number of true negatives, FP is the number of false positives, and FN is the number of false negatives. All the metrics were calculated using the scikit-learn package, and a detailed introduction of the metrics is shown in Table 3.

**Table 3:** Introduction of the metrics

Metric	Description
Accuracy	(TP + TN)/(TP + TN + FP + FN)
Precision	TP/(TP + FP)
Recall	TP/(TP + FN)
F1 score	$2\times (\mathrm{Recall}\times \mathrm{Precision})/(\mathrm{Recall}+\mathrm{Precision})$
AUPRC	Area under the precision-recall curve
AUROC	Area under the receiver operating characteristic

### Reagents

Succinyl-AAPF-pNA peptide (S7388), α-chymotrypsin (C4129),SYPRO orange (S5692) and p-Nph-5’-TMP (T4510) were purchased from Sigma-Aldrich. PolyJet (SL100688) was purchased from SignaGen. CellTiter-Glo reagent (G7571) was purchased from Promega. Nelfinavir Mesylate (NFV, S4282) and Cyclosporin A (CsA, S2286) was purchased from Selleck. Methotrexate (MTX, CSN16844) was purchased from CSNpharm. GSK3 (HY-112921B), ENPP1-IN-1 (E1, HY-129490), ATP (HY-B2176), 2’3’-cGAMP sodium (HY-100564A), Phorbol 12-myristate 13-acetate (PMA, HY-18739) and Ionomycin (HY-13434) were purchased from MedChemExpress. Isopropyl β-D-thiogalactoside (IPTG, A100487) was purchased from Sangon Biotech. Tris-(2-carboxyethyl)-phosphine (TCEP, MB2601) was purchased from Meilun Biotech.

### Peptidyl-prolyl cis-trans isomerase (PPIase) activity assay

CYPA isomerase activities were quantified using a α-chymotrypsin coupled assay in a 96-well plate. The enzymatic reaction mixture (195 μL) contained 50 mmol/L HEPES (pH 8.0), 100 mmol/L NaCl, 1 mg/mL BSA, 1 mg/mL α-chymotrypsin, 2 μmol/L CYPA and 10 μmol/L NFV or CsA. The enzyme reactions were initiated by the addition of 5 μL of 3.2 mmol/L Succinyl-AAPF-pNA peptide dissolved in trifluoroethanol containing 470 mmol/L LiCl. Changes in absorbance due to released *p*-nitroaniline were monitored at 390 nm every 4 s for 6 min at 4 °C using a Tecan Spark microplate reader (Tecan, Mannedorf, Switzerland). This experiment was performed three independent times.

### ENPP1 enzyme activity assay

Evaluation of the ENPP1 activity was carried out with p-Nph-5’-TMP or ATP as the substrate. Enzymatic reactions were performed at 37 °C in a total volume of 100 μL in a clear 96-well plate. The reaction mixture (90 μL) contained 50 mmol/L Tris-HCl (pH 8.5), 130 mmol/L NaCl, 1 mmol/L CaCl_2_, 5 mmol/L KCl, 10 μL ENPP1 cell lysate and different concentration of MTX. The enzyme reactions were initiated by the addition of 10 μL of 1 mmol/L p-Nph-5’-TMP dissolved in deionized water. Changes in absorbance due to released *p*-nitrophenolate were measured at 405 nm every minute for 60 min at 37 °C using a Tecan Spark microplate reader (Tecan, Mannedorf, Switzerland). In the assays where ATP was used as the substrate, the reaction was stopped after 30 min by heating samples at 95 °C for 3 min. The ATP consumption was analyzed by LC-MS/MS (Sciex API-4000). This experiment was performed three independent times.

### Statistical analysis

Statistical analysis for in vitro experiments was done by GraphPad Prism software, version 7.0. Statistical analysis for the model was done by scipy, version 1.2.1. Data are presented as mean ± SD. Differences in the quantitative data between groups were calculated using 2-tailed unpaired *t*-test. *P* < 0.05 was considered to be significant.

## Abbreviations

API, application programming interface; ATR, ATM and RAD3-related; AUPRC, area under the precision-recall curve; BET, bromodomain-containing protein; CETSA, cellular thermal shift assay; cGAS, cyclic GMP-AMP synthase; Cmap, Connectivity Map; CNA1, Calcineurin A alpha; CNBII, Calcineurin B, type II; CP, Compound; CPI scores, the probabilities of whether the compounds show activity towards the potential targets; CP-signatures, compound-induced signatures; CsA, cyclosporine A; CYPA, cyclophilin A; DMSO, dimethyl sulfoxide; ENPP1, ectonucleotide pyrophosphatase/phosphodiesterase-1; GCN, graph convolution network; GEO, Gene Expression Omnibus; GPU, graphics processing unit; HDAC, pan-histone deacetylase; HIV-1, human immunodeficiency virus type 1; IFN-β, interferon beta; IL-2, interleukin-2; KD, knockdown; KD-signatures, gene KD-induced signatures; LINCS, the Library of Integrated Network-Based Cellular Signatures; MNI, the mode-of-action by network identification; MOA, mechanism of action; MTX, methotrexate; NF-AT, nuclear factor of activated T cells; NFV, nelfinavir; PMA, phorbol 12-myristate 13-acetate; PPI, protein-protein interaction; PPIase, peptidyl-prolyl cis-trans isomerase; PRC, the precision-recall curve; RF, random forest; RNA-Seq, RNA sequencing; ROC, the receiver operating characteristic; SSGCN, Siamese spectral-based graph convolutional network; STING, stimulator of interferon genes; TCEP, Tris-(2-carboxyethyl)-phosphine; Tm, melting temperature; Treg, T regulatory cells.

## Supplementary Information

Below is the link to the electronic supplementary material.

Supplementary file1 (PDF 779 kb)

Supplementary file2 (XLSX 19 kb)

## Declarations

We do not compete for commercial interests as defined by Protein & Cell.

The authors declare that all data supporting the findings of this study are available publicly. Both the LINCS phase I L1000 dataset (GSE92742, 2012-2015) and the LINCS phase II L1000 dataset (GSE70138, 2015-2020) were downloaded from the Gene Expression Omnibus (GEO) provided by the Broad Institute. The protein–protein interactions (v11.0) were available at https://string-db.org/. The data set for modeling can be available at https://github.com/boyuezhong/SSGCN/. The code can be freely available at https://github.com/boyuezhong/SSGCN/, under Apache 2.0 license.

M.Z. and H.J. conceived the project. S.Z. designed the experimental validation studies. F.Z. implemented the SGCN model. F.Z. and X.W. conducted computational analysis of transcriptional data. F.Z., X.W., X.L., D.W., Z.F., X.L. and K.C. collected and analyzed the data. R.Y., Z.F., and S.Z. performed *in vitro* experiments. F.Z., X.W., S.Z. and M.Z. wrote the paper. All authors discussed the results and commented on the manuscript.
