# Supplementary material for: Drug target inference by mining transcriptional data using a novel graph convolutional network framework
Source: Protein Cell. 2021 Oct 22;13(4):281–301. doi: 10.1007/s13238-021-00885-0 (PMC8532448; doi:10.1007/s13238-021-00885-0)
Supplement: Supplementary file 1 — Supplementary file1 (PDF 779 kb) [file 13238_2021_885_MOESM1_ESM.pdf]

Supplementary materials

Supplementary information includes: the numbers of KD genes in each KD time (Supplementary Table 1), the predicted results in the PC3 cell line on the LINCS II data (Supplementary Table 2), the relationship between the performance of the model in the test dataset compiled by Pabon et al. and the number of known ligands of the target in the training set (Fig. S1), target space of the test set (Fig. S2), the relationship between protein family and prediction performance of SSGCN model (Fig. S 3), NFV had no effect on calcineurin phosphatase activity (Fig. S4), pipeline of the target-centric prediction of novel ENPP1 inhibitor (Fig. S5), inhibitory activity of 7 test compounds and E1 at 10 μmol/L on ENPP1 (Fig. S6), MTX could not enhance the transcription of *IFNB1* induced by GSK3 in THP-1-derived macrophages (Fig. S7), cytotoxicity determined in THP-1-derived macrophages and MDA-MB-231 cells after 24 h in the present of different concentration of MTX (Fig. S8) , the effect of proportion of negative to positive on model prediction performance (Fig. S9) and the Supplementary Method. The Supplementary Method includes: molecular docking, protein expression and purification, surface plasmon resonance, calcineurin phosphatase activity assay, protein thermal shift assay, cell culture, cellular thermal shift assay, enzyme-linked immunosorbent assay, RNA isolation, cDNA synthesis, and real-time quantitative PCR and cytotoxicity assay.

Computational target inference by mining transcriptional data using a novel Siamese spectral-based graph convolutional network

Supplementary Table 1. The numbers of KD genes in each KD time

Cell lines	The number of genes (96 hours)	The number of genes (120 hours)	The number of genes (144 hours)
PC3	3822	128	1725
A549	3724	0	0
MCF7	3471	0	1837
HT29	3665	0	0
A375	3826	0	0
HA1E	3801	0	0
VCAP	34	4121	0
HCC515	3522	0	0

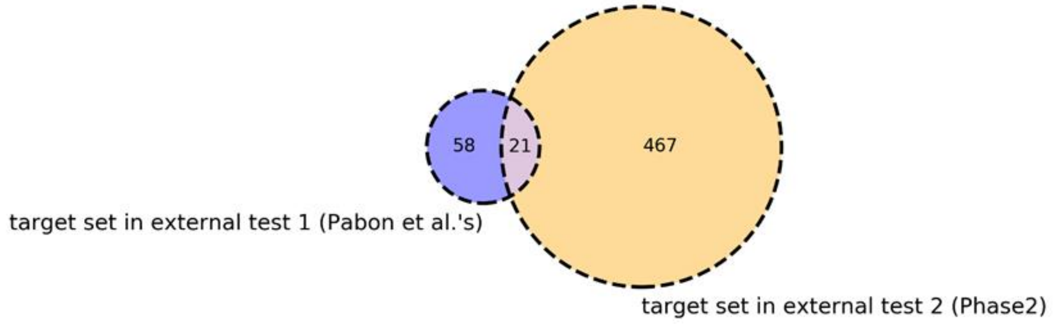

Supplementary Fig.1 Target space of the test set. Digits denoted the target number of the corresponding data set.

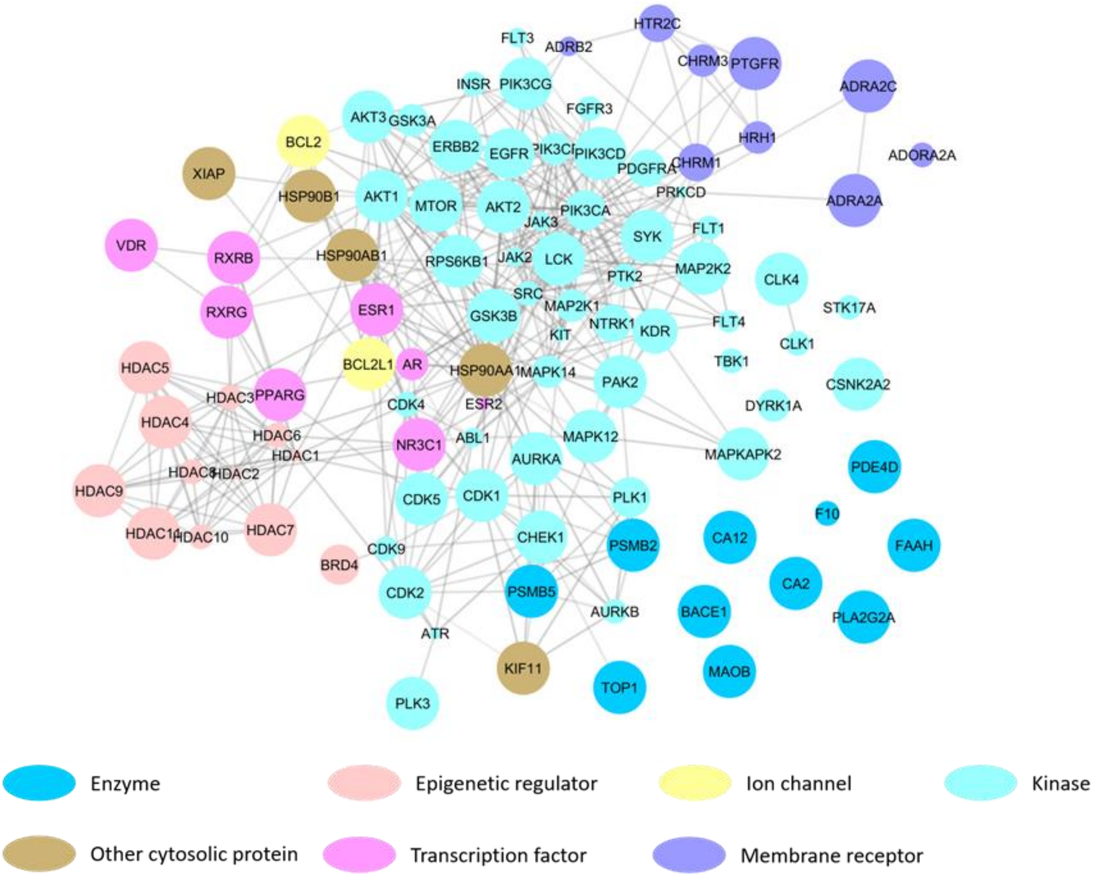

Supplementary Fig. 2 The relationship between protein family and prediction performance of SSGCN model. The top 100 targets giving the best performing predictions are depicted, with the size of sphere indicating the prediction accuracy.

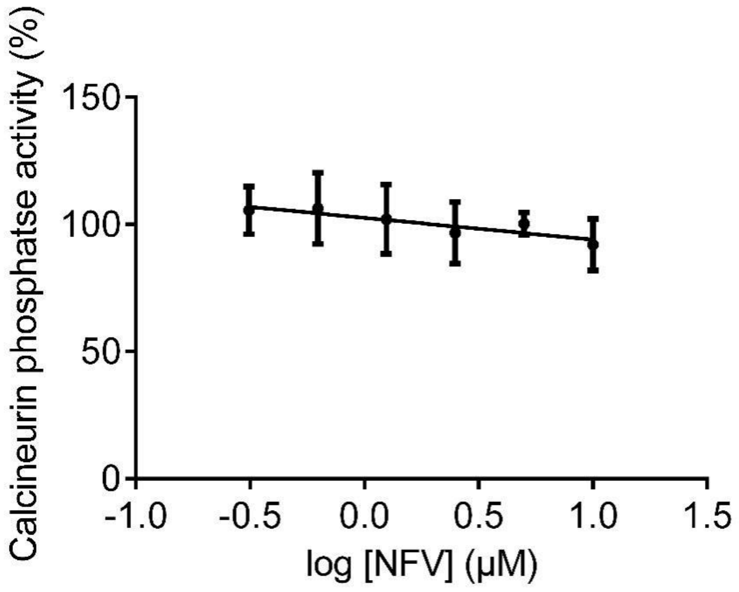

Supplementary Fig. 3 NFV had no effect on calcineurin phosphatase activity. Data are represented as mean ± SD from three independent experiments (n = 3). Source data of this figure are provided in the Supplementary Source data file.

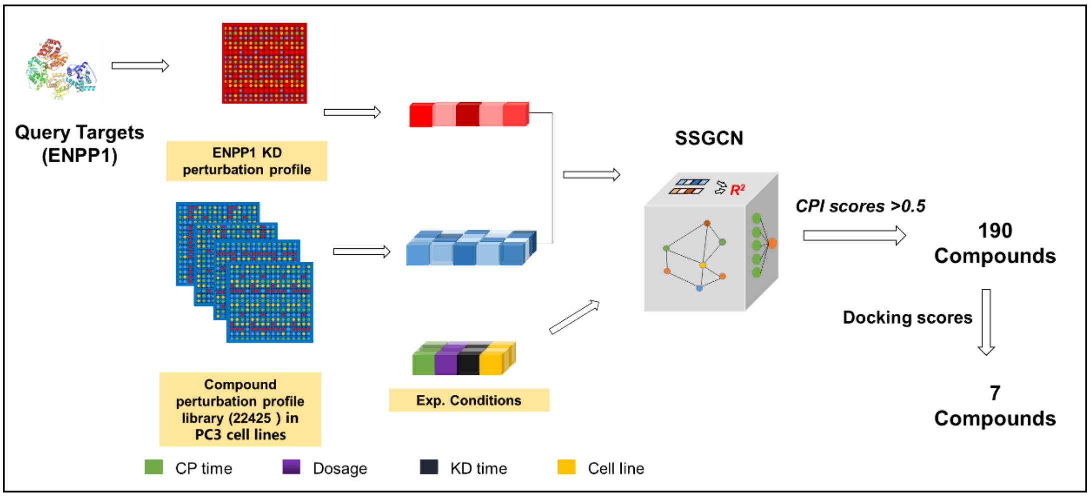

Supplementary Fig. 4 Pipeline of the target-centric prediction of novel ENPP1 inhibitor. R^2 is Pearson R^2 of the compound graph embedding and target graph embedding. The CP time is the duration of compound (CP) treatment and the KD time is the duration of gene knockdown (KD) perturbation. SSGCN is the Siamese spectral-based graph convolutional network. The CPI score is the probabilities of whether the compound interact the protein.

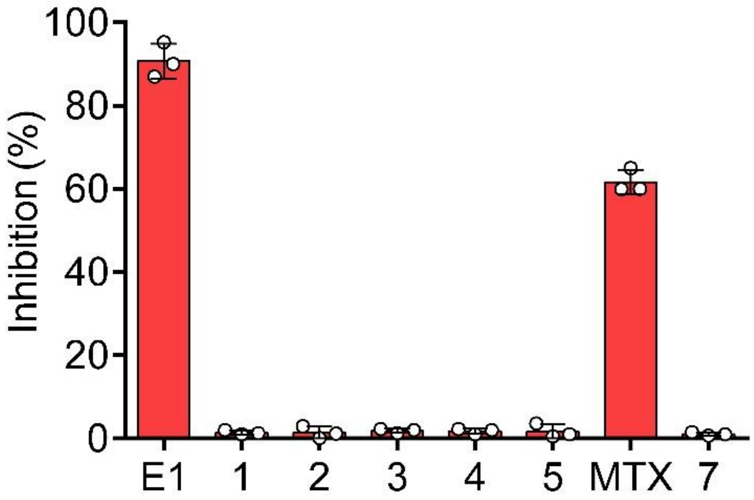

Supplementary Fig. 5 Inhibitory activity of 7 test compounds and E1 at 10 μM on ENPP1. Data are represented as mean ± SD from three independent experiments (n = 3). Source data of this figure are provided in the Supplementary Source data file.

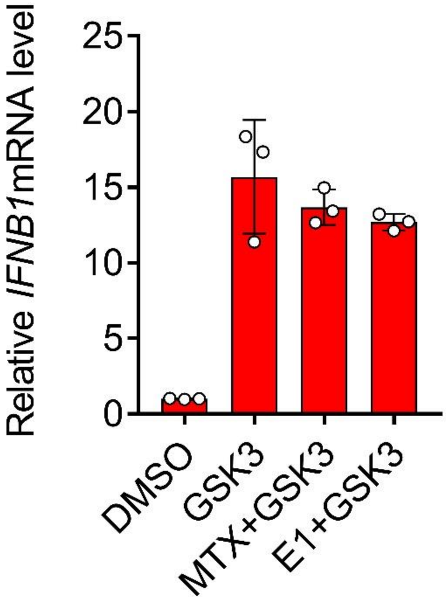

Supplementary Fig. 6 MTX could not enhance the transcription of *IFNB1* induced by GSK3 in THP-1-derived macrophages. THP-1-derived macrophages were treated with MTX (20 μM) or E1 (20 μM), following stimulation with GSK-3 (3 nM) for 24 hours, then cells were collected and subject to RT-qPCR. Data are represented as mean ± SD from three independent experiments (n = 3). Source data of this figure are provided in the Supplementary Source data file.

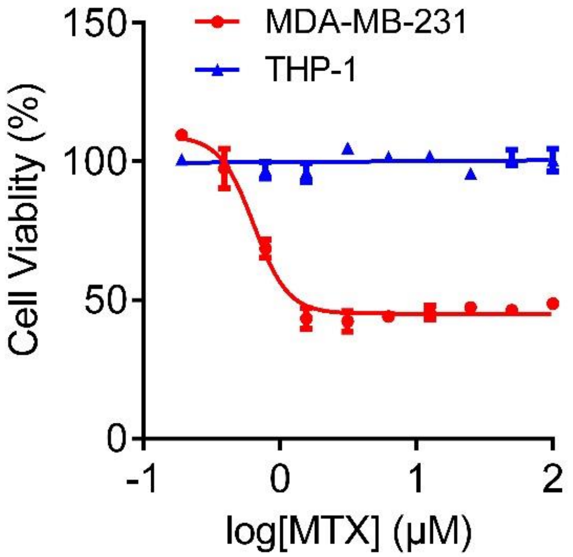

Supplementary Fig. 7 Cytotoxicity determined in THP-1-derived macrophages and MDA-MB-231 cells after 24 hours in the present of different concentration of MTX. Data are represented as mean ± SD from three independent experiments (n = 3). Source data of this figure are provided in the Supplementary Source data file.

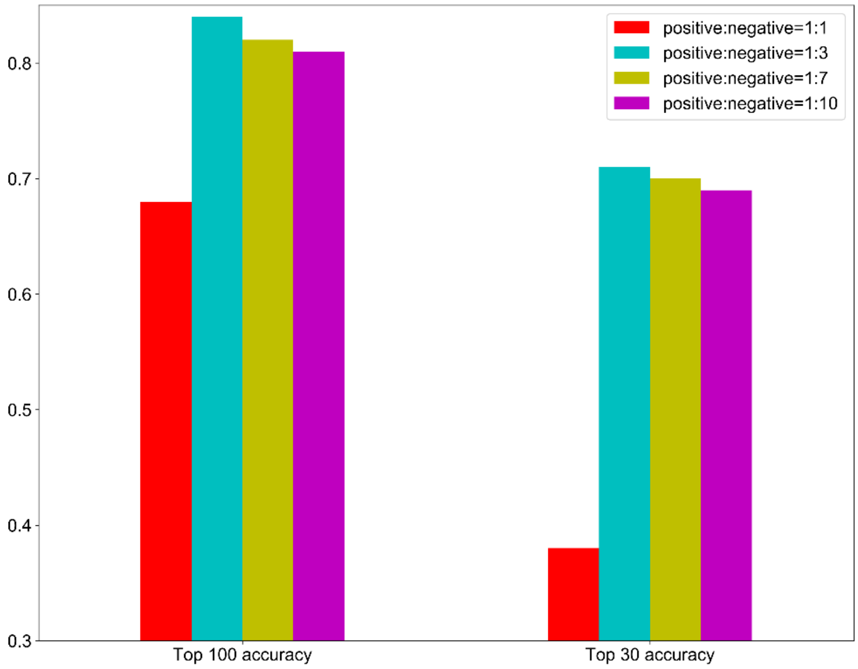

Supplementary Fig. 8 The effect of proportion of negative to positive on model prediction performance.

We explored the performance of the model with positive to negative ratio of

1:1,1:3, 1:7 and 1:10. As shown in Supplementary Fig. 9, the accuracy of our models was not very sensitive to the data proportion, but when balanced data was used, the predictive accuracy of corresponding model dropped significantly. Generally, including more negative samples may compromise the process of training, and prevent the resulted model from producing too high false positive rates in prediction, but there is still controversy about how to set the ratio of negative and positive data to make the model have better generalization ability in practical applications.

SUPPLEMENTARY METHOD

Molecular docking

The human CYPA (PDB ID: 2X2C) and mouse ENPP1 (PDB ID: 4GTW) protein structure were prepared using the Protein Preparation Wizard module in the Maestro program (Maestro, version 9.1; Schrödinger, LLC, New York, NY, 2010) with default parameters. In general, the hydrogen atoms were properly added, bond corrections were applied, all water was removed and the hydrogen bonding network was optimized. Finally, a restrained minimization was performed on the structure of complex using the OPLS3 force field. The low-energy three-dimensional (3D) structures were generated with LigPrep (LigPrep, version 2.4; Schrödinger, LLC, New York, NY, 2010) using OPLS3 force field for docking. The grid file was generated by Glide program. The ligand molecule was identified by manually picking the ligand and the docked ligand was confined to the enclosing box whose center is the centroid of workspace ligand. Docking was performed using Glide software (Glide, version 5.6; Schrödinger, LLC, New York, NY, 2010) using the standard precision (SP) mode with default parameters.

RNA-Seq application protocol:

1. Recommended experiment conditions:

Cell Type	PC3, A549, MCF7, HT29, A375, HA1E, VCAP, HCC515

Duration	6, 24h
Concentration	0-10uM
Replicate Number	≥2

2. Recommended data management:

According to our evaluation, the recommended reference genome version is GRCh38. Abundant bioinformatics tools, such as BioConductor (Huber et al., 2015), can be used to get read counts quantification on gene level. The raw read counts can be transformed into input to our methods as follow:

(1) Convert the count values C_{g_i} of each gene g_i into log values L_{g_i}:

$$L_{g_i} = \log_2(C_{g_i} + 1)$$

(2) Calculate the means $\overline{X}_{g_i,ctrl}$, $\overline{X}_{g_i,trt}$ and variances $S^2_{g_i,ctrl}$, $S^2_{g_i,trt}$ of each gene log values $L_{g_i,ctrl}$, $L_{g_i,trt}$ in control and compound treated groups respectively:

$$\overline{X}_{g_i,ctrl} = \mathrm{mean}\left(L_{g_i,ctrl}\right)$$

$$\overline{X}_{g_i,trt} = \mathrm{mean}\left(L_{g_i,trt}\right)$$

$$S^2_{g_i,ctrl} = \mathrm{var}\left(L_{g_i,ctrl}\right)$$

$$\mathrm{S}^2_{\mathrm{g_i,trt}} = \mathrm{var}\left(\mathrm{L}_{\mathrm{g_i,trt}}\right)$$

(3) Calculate the z-score Z by the following equation:

$$\mathrm{Z} = (\overline{X}_{g_i,trt} - \overline{X}_{g_i,ctrl})/(1.4826 \times \sqrt{(S^2_{g_i,ctrl}/N_{ctrl} + S^2_{g_i,trt}/N_{trt})})$$

N_{ctrl}, N_{trt} are the sample sizes of control and compound treated groups respectively. 1.4826 is a scale factor used in LINCS data(Subramanian et al., 2017).

(4) Retrieve z-scores of landmak genes Z_{lk} from Z, then the Z_{lk} can served as an input to our model for prediction.

3. Practical example:

(1) Download the RNA-Seq raw read count in GSE117942(Guan et al., 2019) from Gene Expression Omnibus.

(2) Extract control samples and Afimoxifene treated sample on MCF7 from the data downloaded in (1).

(3) Perform a data process mentioned in 2 to get Z_{lk}.

(4) Feed Z_{lk} into our trained model to get prediction results.

(5) The reported target ESR2(Hong et al., 2015) of Afimoxifene was ranked 35th position in the results.

Protein expression and purification

The sequence encoding human CYPA was amplified by PCR and inserted into a modified Pet28a vector with His-SUMO tag, this construct was confirmed by DNA sequencing (DNA sequencing data for CYPA are provided in the Supplementary Source data file). The recombinant CYPA protein was expressed in Escherichia coli BL21 (DE3) strain. The cells were grown up at 37 ℃ and induced with 0.4 mM IPTG at 23 ℃ overnight. The fusion protein was purified over a Ni-NTA affinity column. The His-SUMO tag was removed by ULP1 cleavage during dialysis against buffer containing 20 mM MES pH 6.5, 150 mM NaCl, 10 mM imidazole and 1 mM TCEP. The CYPA protein was further purified by gel filtration on superdex 75 increase columns (GE Healthcare, Chicago, USA). The purified protein was concentrated and stored in buffer (20 mM MES pH 6.5, 150 mM NaCl and 1 mM TCEP) at -80 ℃.

Full-length human ENPP1 cDNA was subcloned into a Flag-tagged pcDNA3.1 vector, this construct was confirmed by DNA sequencing (DNA sequencing data for ENPP1 are provided in the Supplementary Source data file). Recombinant human ENPP1 cell lysates were prepared as previously described(Eliahu et al., 2010). In briefly, COS-7 cells were transiently transfected with Flag tagged human ENPP1 using PolyJet according to the manufacturer's protocol. The cells were collected 48 hours after the transfection and disrupted by sonication in buffer containing 100 mM NaCl and 50 mM Tri-HCl. After centrifugation at 300 g for 10 minutes at 4 ℃, the supernatant was aliquoted and stored at -80 ℃ until it was used for enzymatic activity assays.

Surface plasmon resonance (SPR)

Biacore T200 instrument (GE Healthcare, Chicago, USA) was used to perform the SPR binding assays. The CYPA was covalently immobilized onto a CM5 sensor chip using a standard amine-coupling procedure in 10 mM sodium acetate (pH 5.0) with running buffer MES-EP (50 mM MES, pH 6.5, 150 mM NaCl, 0.05% v/v P20). Nelfinavir were serially diluted and injected onto a sensor chip at a flow rate of 30 μl/min for 120 seconds (contact phase), followed by 120 seconds of buffer flow (dissociation phase).

Calcineurin phosphatase activity assay

Calcineurin phosphatase activity was measured using the calcineurin phosphatase activity assay kit (Abcam, ab139461) according to the manufacturer's instructions. In briefly, the enzymatic reaction mixture contained 40 U calcineurin, 0.25 μM calmodulin, 150 μM calcineurin substrate (RII phosphopeptide) and different concentration of NFV. After incubated at 30 ℃ for 30 minutes, the reaction was terminated by addition of 100 μl green assay reagent, and changes in absorbance were measured at 620 nm in 20 minutes using a Tecan Spark microplate reader (Tecan, Mannedorf, Switzerland). This assay was performed in three independent experiments.

Protein thermal shift assay

The thermostability of CYPA was tested using CFX96™ RealTime PCR Detection System (Bio-Rad, Shanghai, China). Nelfinavir were incubated with 2.5 μM CYPA and 5x SYPRO orange in MES buffer (pH 6.5) at different temperature. Fluorescence signal was monitored and collected from 25 to 95 ℃ within 45 minutes.

Cell culture

Human myeloid leukemia mononuclear cells (THP-1) (ATCC, TIB-202) were cultured in RPMl Medium 1640 (Gibco, 61870-036) supplemented with 10% FBS

(Gibco, 10099-141) and 0.05 mM β-mercaptoethanol (Gibco, 21985). THP-1 cells were differentiated into macrophage-like cells (THP-1-derived macrophages) by incubation in the presence of PMA (100 nM) for 48 hours. COS-7 cells (Cell Bank of the Chinese Academy of Sciences, SCSP-508), MDA-MB-231 cells (ATCC, HTB-26) were cultured in RPMl Medium 1640 (Gibco, 61870-036) supplemented with 10% FBS (Gibco, 10099-141). The mouse macrophage cell line (RAW 264.7) (ATCC, TIB-71) and 293T cells (Cell Bank of the Chinese Academy of Sciences, SCSP-502) were cultured in Dulbecco's Modified Eagle's medium (DMEM, Gibco, 10569-010) supplemented with 10% FBS (Gibco, 10099-141). All cells were incubated at 37 ℃ under 5% (v/v) CO_2 atmosphere.

Cellular thermal shift assay (CETSA)

Cellular thermal shift assay was conducted according to the protocol as previously described(Molina et al., 2013). 293T cells transiently transfected with Flag-ENPP1 for 48 hours were collected and lysed in 20 mM Tris-HCl (pH 7.5), 150 mM NaCl and 1% Triton X-100. Then, 50 μM MTX or DMSO was added to the supernatant and incubated at 25 ℃ for 30 minutes. After denaturing at various temperatures for 3 minutes on temperature gradient PCR instrument (Eppendorf, Hamburg, Germany), samples were centrifuged at 20000 g for 30 minutes at 4 ℃ and the supernatants were analyzed by Western blot. All samples containing 1 × loading buffer (Beyotime, P0015) were denatured at 100 ℃ for 5 minutes and separated by 8% SDS-PAGE, and then transferred to nitrocellulose membranes. Membranes were blocked with Blocking buffer (Beyotime, P0252) and then incubated with anti-Flag (Cell Signaling Technology, 14793) or anti-GAPDH (Cell Signaling Technology, 5174) primary antibodies overnight at 4 ℃, followed by incubation with HRP-conjugated anti-rabbit secondary antibody (Promega, W4011) for 1 hours at room temperature. Lastly, the immune complexes were detected with ECL kit (Meilun, MA0186) and visualized using ChemiDoc™ XRS system from Bio-Rad (Shanghai, China). This assay was performed in three independent experiments.

Enzyme-linked immunosorbent assay (ELISA)

ELISA was performed on cell culture supernatants from indicated cells for the detection of human IFNB1 (R&D Systems, DY814-05), mouse IFNB1 (R&D Systems, DY8234-05) and human IL-2 (Absin, abs510001) according to the manufacturer's instructions. In briefly, added 100 μl of culture supernatant or standards in 96-well microplate coated with capture antibody and incubated 2 hours at room temperature; aspirated each well and washed with wash buffer three times, then added 100 μl of the working dilution of detection antibody to each well and incubated 2 hours at room temperature; aspirated each well and washed with wash buffer three times, then added 100 μl of the working dilution of streptavidin-HRP to each well and incubated 20 minutes at room temperature (avoided placing the plate in direct light); aspirated each well and washed with wash buffer three times, then added 100 μl of substrate solution to each well and incubated 20 minutes at room temperature (avoided placing the plate in direct light), following add 50 μl of stop solution to each well and gently tapped the plate to ensure thorough mixing; then immediately measured the optical density of each well at 450 nm (reference wavelength: 570 nm). Lastly, a standard curve was created and the concentration of all samples were calculated according to the standard curve.

RNA isolation, cDNA synthesis, and real-time quantitative PCR (RT-qPCR)

Total RNA was isolated from cells using RNA extraction reagent (Vazyme, R401-01). cDNA was synthesized using the HiScript ⅡQ RT SuperMix (Vazyme, R223-01) according to manufacturer's instructions. RT-qPCR was performed using ChamQ SYBR qPCR Master Mix (Vazyme, Q331-02) in CFX96^{TM} RealTime PCR Detection System (Bio-Rad, Shanghai, China). The profile of thermal cycling consisted of initial denaturation at 95 ℃ for 30 seconds, and 40 cycles at 95 ℃ for 5 seconds and 60 ℃ for 30 seconds. All the primer sequences used in this study are as follows: mouse *Actb* forward: tgagctgcgttttacaccct, mouse *Actb* reverse: gccttcaccgttccagtttt; mouse

Ifnb1 forward: gtcctcaactgctctccact, mouse *Ifnb1* reverse: cctgcaaccaccactcattc. mouse *Cxcl10* forward: atcatccctgcgagcctatcct, mouse *Cxcl10* reverse: gaccttttttggctaaacgctttc; mouse *Il6* forward: acaagtcggaggcttaattacacat, mouse *Il6* reverse: ttgccattgcacaactcttttc; human *ACTB* forward: catgtacgttgctatccaggc, human *ACTB* reverse: ctccttaatgtcacgcacgat; human *IFNB1* forward: cagcatctgctggttgaaga, human *IFNB1* reverse: cattacctgaaggccaagga; human *CXCL10* forward: ccacgtgttgagatcattgct, human *CXCL10* reverse: tgcatcgattttgctcccct; human *IL6* forward: ttcggtccagttgccttctc, human *IL6* reverse: tacatgtctcctttctcagggc.

Cytotoxicity assay

Cell viability was measured using the CellTiter-Glo reagent according to the manufacturer's instructions. THP-1 cells were pre-seeded in 96-well plate (3000 cells/well) in the presence of PMA (100 nM) for 48 h, then serially diluted compounds were added. After 24 hours, ATP was measured using CellTiter-Glo Viability Assay. This assay was performed in three independent experiments.

Supplementary References

Eliahu, S., Lecka, J., Reiser, G., Haas, M., Bigonnesse, F., Lévesque, S.A., Pelletier, J., Sévigny, J., and Fischer, B. (2010). Diadenosine 5′,5″-(boranated)polyphosphonate analogues as selective nucleotide pyrophosphatase/phosphodiesterase inhibitors. J Med Chem 53, 8485-8497.

Guan, J., Zhou, W., Hafner, M., Blake, R.A., Chalouni, C., Chen, I.P., De Bruyn, T., Giltnane, J.M., Hartman, S.J., Heidersbach, A., et al. (2019). Therapeutic Ligands Antagonize Estrogen Receptor Function by Impairing Its Mobility. Cell 178, 949-963.e918.

Hong, H., Branham, W.S., Ng, H.W., Moland, C.L., Dial, S.L., Fang, H., Perkins, R., Sheehan, D., and Tong, W. (2015). Human sex hormone-binding globulin binding affinities of 125 structurally diverse chemicals and comparison with their binding to androgen receptor, estrogen receptor, and α-fetoprotein. Toxicol Sci 143, 333-348.

Huber, W., Carey, V.J., Gentleman, R., Anders, S., Carlson, M., Carvalho, B.S., Bravo, H.C., Davis, S., Gatto, L., Girke, T., et al. (2015). Orchestrating high-throughput genomic analysis with Bioconductor. Nature Methods 12, 115-121.

Molina, D.M., Jafari, R., Ignatushchenko, M., Seki, T., Larsson, E.A., Dan, C., Sreekumar, L., Cao, Y., and Nordlund, P. (2013). Monitoring drug target engagement

in cells and tissues using the cellular thermal shift assay. Science 341, 84.
Subramanian, A., Narayan, R., Corsello, S.M., Peck, D.D., Natoli, T.E., Lu, X.D., Gould, J., Davis, J.F., Tubelli, A.A., Asiedu, J.K., et al. (2017). A Next Generation Connectivity Map: L1000 Platform and the First 1,000,000 Profiles. Cell 171, 1437-1452.
